# Genome-Wide Analysis of AP2/ERF Superfamily Genes in Contrasting Wheat Genotypes Reveals Heat Stress-Related Candidate Genes

**DOI:** 10.3389/fpls.2022.853086

**Published:** 2022-04-13

**Authors:** Manu Maya Magar, Hui Liu, Guijun Yan

**Affiliations:** UWA School of Agriculture and Environment, The UWA Institute of Agriculture, The University of Western Australia, Crawley, WA, Australia

**Keywords:** qRT-PCR, transcription factor, abiotic stress, gene expression, heat tolerance

## Abstract

The AP2/ERF superfamily is one of the largest groups of transcription factors (TFs) in plants, which plays important roles in regulating plant growth and development under heat stress. A complete genome-wide identification, characterization, and expression analysis of AP2/ERF superfamily genes focusing on heat stress response were conducted in bread wheat. This study identified 630 putative AP2/ERF superfamily TF genes in wheat, with 517 genes containing well-defined AP2-protein domains. They were classified into five sub-families, according to domain content, conserved motif, and gene structure. The unique genes identified in this study were 112 TaERF genes, 77 TaDREB genes, four TaAP2 genes, and one TaRAV gene. The chromosomal distribution analysis showed the unequal distribution of TaAP2/ERF genes in 21 wheat chromosomes, with 127 pairs of segmental duplications and one pair of tandem duplication, highly concentrated in TaERF and TaDREB sub-families. The qRT-PCR validation of differentially expressed genes (DEGs) in contrasting wheat genotypes under heat stress conditions revealed that significant DEGs in tolerant and susceptible genotypes could unequivocally differentiate tolerant and susceptible wheat genotypes. This study provides useful information on TaAP2/ERF superfamily genes and reveals candidate genes in response to heat stress, which forms a foundation for heat tolerance breeding in wheat.

## Introduction

Wheat (*Triticum aestivum* L.) is a globally important cereal that contributes nearly 20% of total human calorie consumption (Appels et al., 2018). However, wheat production is threatened by the constant increase in global temperature, which is predicted to rise by 2°C–5°C by 2050 (FAO et al., 2019; NCEI and NOAA National Centers for Environmental Information, 2021). The severity of heat damage depends on crop growth stage, duration, and frequency of heat stress (HS). The negative effect of HS can be minimized by understanding the heat tolerance mechanism and developing heat resilient wheat varieties (Ali et al., 2020). The conventional breeding approach has developed some heat-tolerant varieties, but the genetic and molecular–physiological basis of heat tolerance is still largely unknown (Driedonks et al., 2016). Investigation of gene responses under HS can facilitate the understanding of heat tolerance mechanism.

Heat-responsive genes can be studied through relative gene expression profiles under HS. Expression of HS-related traits involves activation of molecular networks by transcription factors (TFs), expression of heat-responsive genes, and production of metabolites (Singh et al., 2019). The expression of a gene under stress is regulated by TFs, which interact with a specific sequence in the promoter to control its transcription. The TF families HSF, AP2/ERF, NAC, MYB, WRKY, GRF, ARF, bHLH, SBP, HD-ZIP, b-ZIP, and Zn-finger TF genes have been widely reported as abiotic stress-responsive TF family genes in rice and maize (Begcy et al., 2019; Qian et al., 2019; Liu G. et al., 2020; Liu H. et al., 2020; Wang et al., 2020).

The APETALA 2/ethylene-responsive factor (AP2/ERF) superfamily is one of the most diverse families of plant TFs, which is known for its regulatory role in biotic/abiotic stress-, growth-, and development-related gene expression (Debbarma et al., 2019). The AP2/ERF genes can be classified into AP2, ERF, DREB, RAV, and others based on the presence and specificity of domains (Sakuma et al., 2002; Nakano et al., 2006). The AP2 domain was first identified in *Arabidopsis* as a regulator in the flower development (Jofuku et al., 1994), and the ERF domain was first identified in tobacco as an ethylene-responsive TF (Ohme-Takagi and Shinshi, 1995). Similarly, RAV genes were first identified in *Arabidopsis* as full-length cDNAs encoding B3-like and AP2 domain (Kagaya et al., 1999) and were reported to involve in ethylene response (Alonso et al., 2003) and brassinosteroid response (Hu et al., 2004). The heat-induced expressions of AP2/ERF family genes were reported in various crop species, such as *Arabidopsis* (Chen et al., 2012), chrysanthemum (Hong et al., 2009), rice (Matsukura et al., 2010), sunflower (Najafi et al., 2018), maize (Gu et al., 2019), tomato (Deng et al., 2020), and orchardgrass (Xu et al., 2020). In wheat, heat-responsive probes obtained from GeneChip Wheat Genome Array and encoding DREB genes were induced under HS (Qin et al., 2008) and DREB genes overexpressed in transgenic *Arabidopsis* showed heat tolerance (Niu et al., 2020).

Availability of wheat reference genome sequence in the public domain has facilitated the rigorous genome wide identification and functional characterization of genes under different stress conditions. In wheat, genome-wide identification and analysis of few gene families have been reported, such as of NAC genes under drought stress (Guerin et al., 2019), DNA binding with one finger (Dof) genes under salt and drought stress (Fang et al., 2020), Hsf genes under drought and HS (Ye et al., 2020), MIKC-type MADS-box genes (Schilling et al., 2020), and caseinolytic protease B (CLPB) proteins (Erdayani et al., 2020) in wheat under HS. In regard to genome-wide expression analysis of AP2/ERF superfamily genes in wheat, previous studies include genome-wide identification of TaERF family genes and expression under heat, drought, and salinity in the wheat cv. Chinese Spring (Riaz et al., 2021), genome-wide identification of TaAP2 family genes and overexpression in transgenic *Arabidopsis* (Zhao et al., 2019), and genome-wide identification of TaDREB family genes and overexpression in transgenic *Arabidopsis* under heat, drought, and salt stress (Niu et al., 2020). TaAP2 family genes are found to regulate organ development in transgenic *Arabidopsis* (Zhao et al., 2019). No functional analysis of TaRAV family genes was reported in wheat so far. Overall, the comprehensive identification, expression analysis of all four families of TaAP2/ERF superfamily TF genes, and their response to HS in contrasting wheat genotypes have not yet been reported. Therefore, the objectives of this study were to identify the AP2/ERF genes present in the whole wheat genome, classify and characterize the identified genes, and analyze their expressions in contrasting wheat genotypes to identify key candidate genes under HS conditions.

## Materials and Methods

### Identification, Classification, and Characterization of AP2/ERF Genes in Wheat

The wheat reference genome and protein sequences were downloaded from the International Wheat Genome Sequencing Consortium website (IWGSC RefSeq v1.1; Appels et al., 2018).^1^ The latest Hidden Markov Model (HMM) profile of AP2/ERF protein domain (PF00847) was downloaded from ProIsomerase family domain model (Pfam) database^2^ (El-Gebali et al., 2019), used as a query sequence to HMM search using HMMER3.1b2 software^3^ in the wheat reference protein sequence version 1.1, with an E-value threshold of 0.1. The genomic locations of these sequences were identified based on their IWGSC annotations, and NCBI CD-search software was used to check the presence of the AP2 domain,^4^ and the sequences without well-defined AP2-domain were discarded. Biochemical properties (molecular weight, theoretical isoelectric point, amino acid count, and GRAVY values) of TF genes were determined by online protein analysis software EXPASy.^5^ The domain sequence of a single AP2 domain containing genes was retrieved from the Pfam database and analyzed for the presence of conserved amino acids at 14th and 19th position to categorize them into ERF and DREB sub-family genes using Geneious Prime.^6^

### Analysis of Phylogeny, Conserved Motifs, and Gene Structure

The multiple alignments of full-length protein sequences of TaAP2/ERF genes with at least one well-defined AP2 domain were performed by MUSCLE with default parameters. The aligned sequences were used to construct a phylogenetic tree using the neighbor-joining method with bootstrap tests of 1,000 replications in MEGAX (Hall, 2013) and visualized using Interactive Tree of Life (iTOL).^7^

The analysis of conserved motif distribution in full-length protein sequence was done using MEME online tool^8^ in each group of four TaAP2/ERF TF gene families (TaRAV, TaAP2, TaDREB, and TaERF). The parameters used in motif analysis were the maximum number of motifs 15 with motif length set between 5 and 200 amino acids. The gene annotation information was examined using Gene Structure Display Server (GSDS)^9^ to predict gene structure, and the hierarchal diversity of TaAP2/ERF genes was analyzed using MEGAX software with 1,000 bootstrap repeats. The final visualization was done in TBtools software.^10^

### Chromosome Locations and Gene Duplication Analysis

The chromosomal location of TaAP2/ERF genes was determined using genome annotation file (.gff3 file) in IWGSC RefSeqv1.1 database,^11^ and MapChart 2.32^12^ was used for mapping TaAP2/ERF genes on different chromosomes of wheat. In order to identify gene duplications, the similarity and identity matrix of TaAP2/ERF genes were calculated in the sequence identity and similarity program^13^ using BLOSUM62 matrix against the length of the largest sequence. The gene duplication events of TaAP2/ERF genes were investigated based on the following two common criteria referred as segmental duplication by: (1) the alignment covers >90% of the longer gene and (2) the alignment has >90% similarity of identity (Shrestha et al., 2021). The two or more segmental duplicated genes, which were located on the same chromosome, one following the other within 100-Kb region, are considered as tandem duplicated genes (Li et al., 2020; Shrestha et al., 2021).

### Plant Materials and Stress Treatment

**Figure 1:**
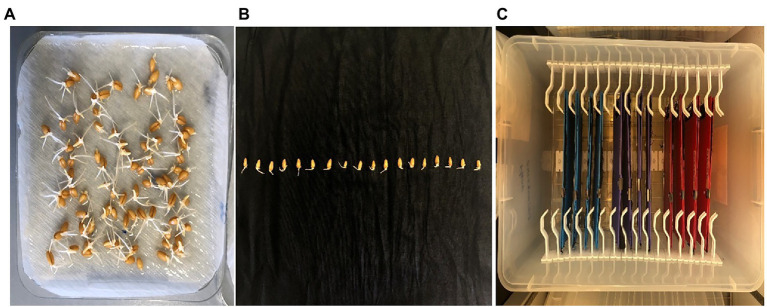
Morphological assessment of wheat seedling under control (25 ± 1°C) and heat stress (37 ± 1°C). **(A)** Germinated wheat seeds; **(B)** germinated seeds transferred to black calico cloth; and **(C)** incubation box with germinated seeds holding folders kept in vertical position.

Heat treatment experiment was conducted in a completely randomized design with three biological replications. Seeds of four wheat genotypes, Perenjori, W156, Brazil 32, and Yitpi, were obtained from the Australian Winter Cereals Collection. Perenjori and W156 were heat tolerant, Brazil 32 and Yitpi were heat susceptible, according to a previous report (Alsamadany, 2016). The seeds were surface-sterilized by washing with 100% ethanol for 30 s followed by washing with 1% sodium hypochlorite for 10 min with constant stirring and rinsing three times with sterile distilled water. The surface-sterilized seeds were germinated under room temperature (25 ± 1°C) under dark conditions for 36 h. The seeds with protruded coleoptile and radicle were selected and transferred to plastic folders between the sterile and wet calico-black cloth. Once the germinated seeds were placed between the clothes, it was clipped with folder clips to ensure that seeds do not move. These folders were then placed half (12 folders) at room temperature (25 ± 1°C) as control and half (12 folders) at 35 ± 1°C for HS treatment in a self-designed box with clips holding the folder upright with one end dipped in sterile distilled water (Figure 1). The seedlings after 3 days of heat treatment were used for morphological trait (root depth) measurement followed by a sampling of the whole seedling (snap frozen) and stored at −80°C for RNA extraction.

### RNA Isolation, cDNA Synthesis, and Gene Expression Analysis

The expression profile of TaAP2/ERF genes was analyzed based on the RNA sequence data available in the wheat expression database.^14^ The expression profile of these genes was monitored in different tissues (leaves, shoots, roots, and spikes) under seedling, vegetative, and reproductive stages for their response to abiotic stress based on their expression reported in Chinese Spring, Azhurnaya, and other wheat genotypes. The expression profile (transcript per million/tpm value) of genes under abiotic conditions was used to generate a heatmap in R software. The genes with relatively higher expression under abiotic stresses were selected, and 2 Kb promoter sequence of the selected gene was analyzed for the presence of cis-regulatory elements using the CARE program^15^ in the PlantCARE database (Song et al., 2019). Further, the individual mRNA sequence of these genes was extracted from the IWGSC reference sequence^16^ was used to design gene-linked primers spanning the exons using primer3 in Geneious Prime.^17^

The whole seedlings were snap-frozen in liquid nitrogen, stored at −80°C, and used for total RNA extraction. RNA was extracted from 24 samples (4 genotypes × 3 replications × 2 treatments) using RNeasy Plant Mini Kit (Qiagen) with an on-column DNase digestion with RNase-free DNase I (Qiagen). The extracted total RNA was quantified using Nanodrop spectrophotometer ND-1000, and the concentration was also verified by using Qubit RNA BR Assay kit in Qubit 3.0 Fluorometer (Invitrogen by life technologies Ref: Q33216). The integrity of RNA was tested using gel electrophoresis with 5 μl RNA in 1.5% agarose gel. The quantified RNA was used for the cDNA synthesis using SensiFAST cDNA Synthesis Kit from Meridian Bioscience (BIO-65054) following the kit protocol with 1 μg RNA in 20 μl reaction volume. The gene-specific primers were designed using Geneious Prime for the 24 representative genes and were validated by PCR amplification. The primers that amplified a single specific band within a defined range (100–200 bp) were selected for further qPCR analysis. The primers were also checked for the single peak in the melting curve to avoid primer-dimer and genomic DNA contamination influencing the assay.

Real-time quantitative PCR (RT-qPCR) was carried out in 10 μl reaction volume containing 25 ng cDNA, 8 μM gene-specific primer mix, 5 μl 2x SensiFAST SYBR Lo-ROX mix and water to make up the volume to 10 μl, on Applied Biosystem 7500/7500 Fast Real-Time PCR System. The protocol was carried out by initial denaturation at 95°C for 30 s, denaturation at 95°C for 3 s, primer annealing at specific annealing temperature for 30 s for 40 cycles followed by default melt curve analysis. Each sample was analyzed in three biological replications and two technical repeats. The β-actin was used as a housekeeping gene for qPCR reaction, and the expression values were calculated by using the 2^−∆∆CT^ method (Livak and Schmittgen, 2001). The expression values obtained were used to calculate the fold change (FC) for each gene by comparing the expression under treatment over control for both tolerant and susceptible genotypes, and Log_2_FC values were used to analyze the pattern of gene regulation. The genes were considered significantly up- or down-regulated when their Log_2_FC was ≥1 and ≤−1, respectively (Li et al., 2020).

## Results

### Identification of AP2/ERF Genes in Wheat

A total of 630 putative AP2/ERF superfamily transcription factor (TF) genes were identified based on genome-wide HMM search using HMM model of AP2 domain (Pfam Id; PF00847) in the wheat genome (Supplementary Table 1). NCBI-domain search confirmed 517 TaAP2/ERF TF genes with well-defined AP2-protein domains, a characteristic domain for AP2/ERF superfamily TF genes and classified them into three families: TaERF/DREB (425 genes with a single AP2 domain), TaAP2 (66 genes with two AP2 domains), and TaRAV (26 genes with a single AP2 domain and a single B3 domain).

**Figure 2:**
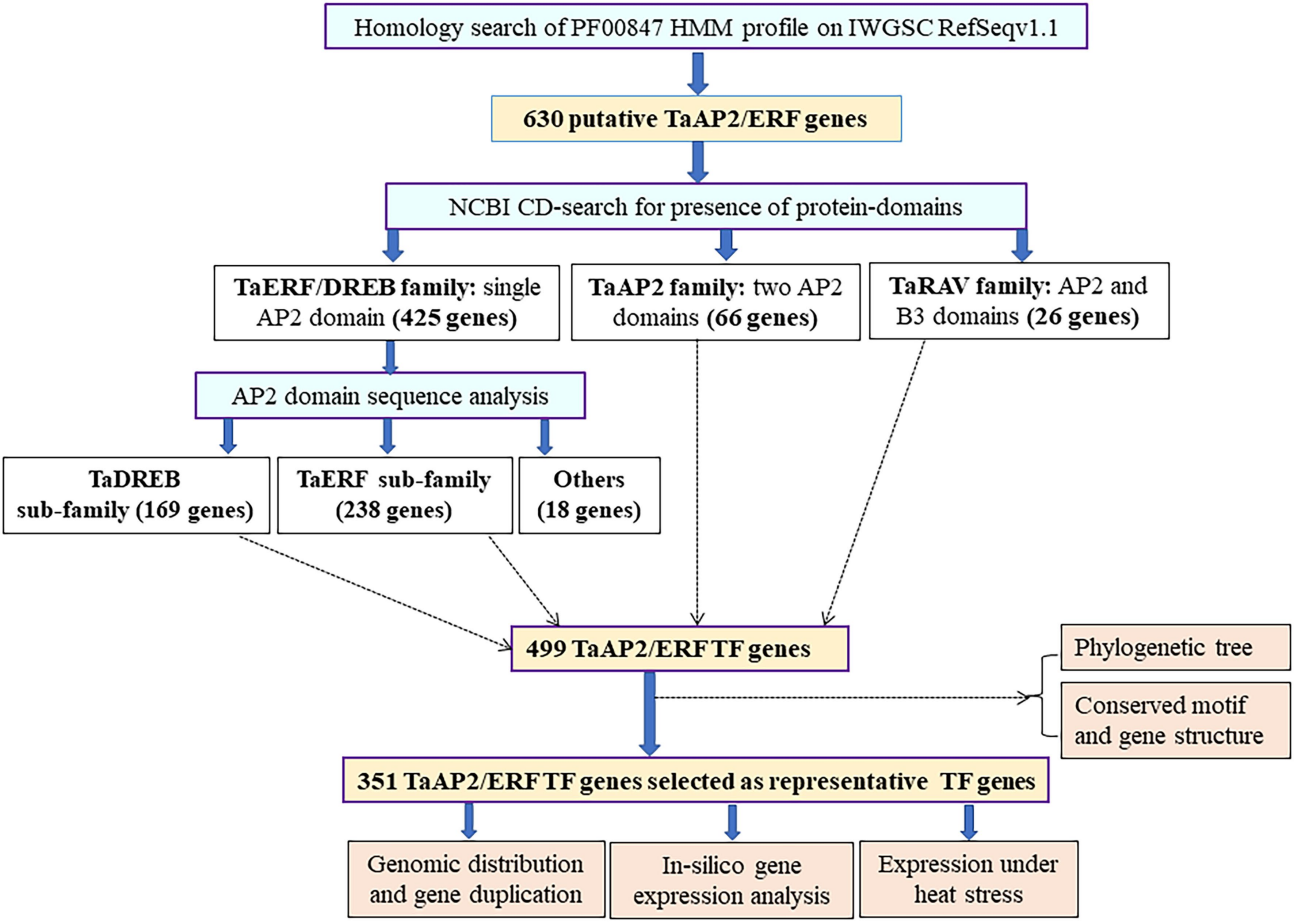
Flowchart showing procedure followed in the TaAP2/ERF TF gene classification. The number of genes used in the major bioinformatic analysis is denoted by a bold letter inside the purple boxes with yellow background, the number of genes in gene families and sub-families are shown in bold letters in black boxes, and analysis conducted in those genes is given in black boxes with brown background.

The domain sequence 423 TaERF/DREB family genes were obtained from Pfam database, and their multiple alignment revealed presence of three amino acids “WLG” conserved in most of the genes, except 25 genes which have “WLG” replaced with “WIS,” “FLG,” and “YLG” in one gene each, “WID” in three genes, “GLG” in four genes; and “WIG” in 15 genes (Supplementary Figures 1–3). The domain sequence analysis classified them into (1) TaDREB sub-family showing conserved amino acid at 14th (V: Valine) and 19th (E: Glutamic acid) positions (169 genes with both or at least one amino acid conserved; Supplementary Figure 1); (2) TaERF sub-family showing conserved amino acid at 14th (A: Alanine) and 19th (D: Aspartic acid) positions (238 genes with both or at least one amino acid conserved; Supplementary Figure 2); and (3) other sub-family (18 genes; Supplementary Figure 3). The stepwise procedures followed to classify the genes into different family and sub-families are summarized in Figure 2.

Subsequent analysis of biochemical properties of proteins encoded by TaAP2/ERF genes including 499 genes comprising of TaERF (238), TaDREB (169), TaAP2 (66), and TaRAV (26) with EXPASy online tool showed that the length of protein ranged from 847 amino acids (*TraesCS2A02G514200.1*) to 119 amino acids (*TraesCS1D02G140900.1*, *TraesCS1A02G187900.1*, and *TraesCS1B02G158400.1*). Similarly, molecular weight ranged from 90.399 KDa in *TraesCS2A02G514200.1* to 12.619 KDa in *TraesCS1A02G187900.1*. Theoretical isoelectric point ranged from 4.39 in *TraesCS5A02G071300.1* to 11.9 in *TraesCS5B02G311300.1*. We also measured the GRAVY values of each gene, which ranged from a positive value of 0.191 in *TraesCS5A02G310100.1* to a negative value of −1.149 in *TraesCS5D02G447200.1* (Supplementary Table 2).

### Analysis of Phylogeny, Conserved Motif, and Gene Structure of AP2/ERF Genes in Wheat

**Figure 3:**
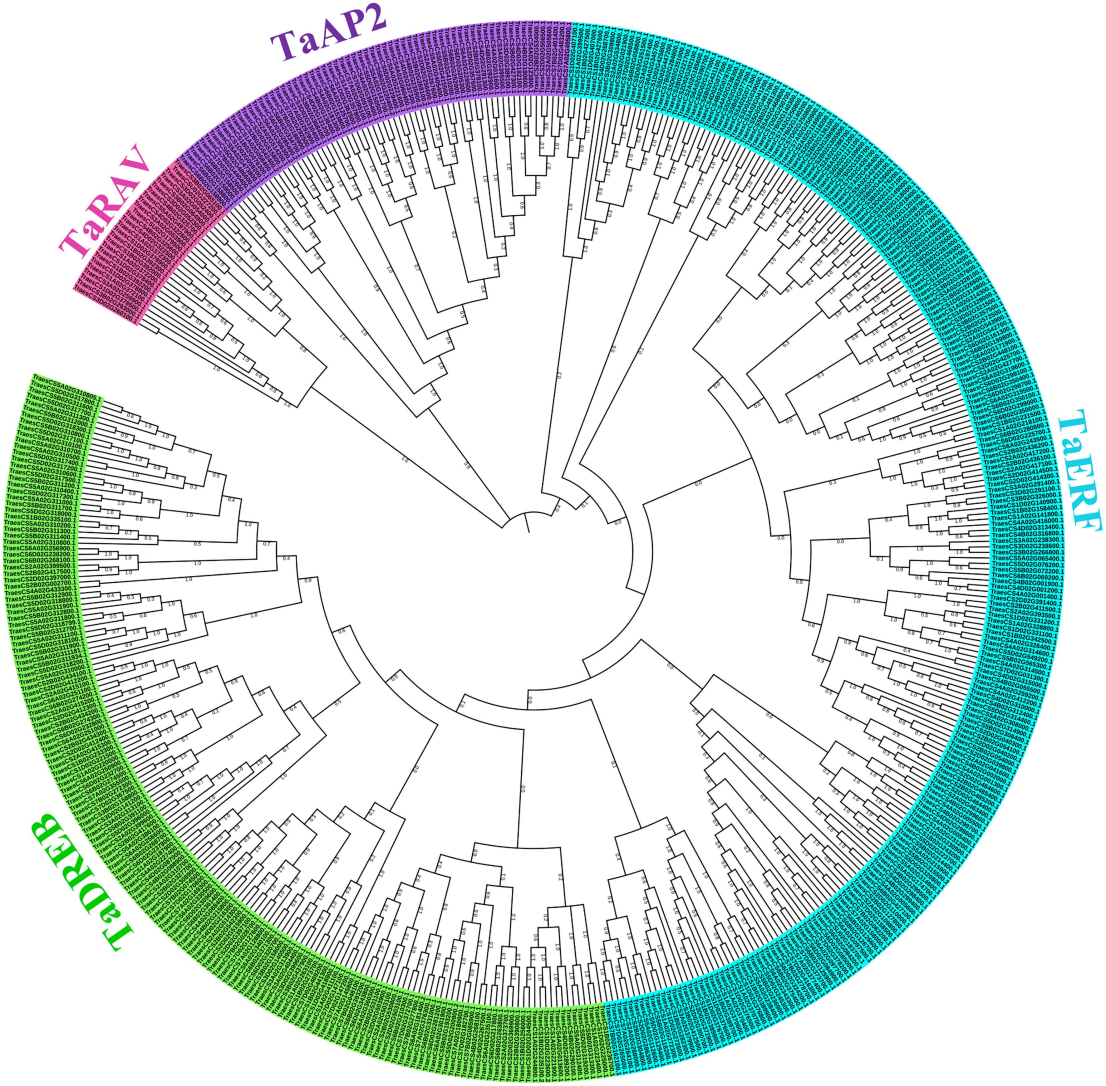
Phylogenetic tree of 499 AP2/ERF TF genes in wheat. The unrooted neighbor-joining tree was constructed using a full-length protein sequence of 499 TaAP2/ERF TF genes in MEGAX software with a bootstrap test of 1,000 replications, which classified these genes into distinct four clusters: TaRAV family (26 TF genes in pink color), TaAP2 family (66 TF genes in purple color), TaERF sub-family (238 TF genes in light blue color), and TaDREB sub-family (169 TF genes in green color).

Phylogenetic analysis of 499 TaAP2/ERF TF genes using MEGAX showed three major clusters with TaRAV, TaAP2, and TaERF/DREB family TF genes. The TaERF/DREB cluster was further branched into the TaERF sub-family with 238 TF genes and TaDREB sub-family with 169 TF genes (Figure 3). The simultaneous branching of three gene families and late branching of TaDREB suggested that TaDREB sub-family genes were evolved later than the other three gene families.

The gene structure analysis with GSDS online server was helpful to better understand the structural diversity of TaAP2/ERF family. The TaAP2 family has four intronless genes with single CDS (exon), one gene with the maximum number of introns (10 introns), and 10 genes with the highest number of exons (10 exons). The TaDREB family genes have 139 intronless genes, two genes with the highest number of introns (three introns), 146 genes with a single exon, and two genes with the highest number of exons (four exons). The TaERF family genes have 174 intronless genes, three genes with the highest number of introns (three introns), 179 genes with a single exon, and one gene with the highest number of exons (three exons). The TaRAV family genes have 24 intronless genes and two genes with single introns and the highest number of exons are two, present in two genes as shown in Supplementary Figures 4A–D.

The conserved motif analysis shows that the number of motif present per gene ranged from 4 to 11 in 169 TaDREB genes. The longest motif 11 (45 amino acids) is present in 11 sites, whereas motifs 2, 4, and 10 are the shortest motif with 12 amino acids. The motif 1 (26 amino acid long) and motif 4 (12 amino acid long) are most frequently present in 169 sites, representing all members of the TaDREB family, while motif 14 (43 amino acid long) is least frequent present only in six sites as shown in Supplementary Figure 4A. Among 15 motifs detected in 238 TaERF genes, the number of motif present per gene ranged from 2 to 8. The longest motif 15 (84 amino acid long) is present in the least number of sites (six sites), and motif 7 (22 amino acid long) is the shortest motifs present in 36 sites. The motif 1 (32 amino acid long) is most frequent, present in all the members (238 sites) of TaERF family genes. Here, we have observed the different positions of the same motifs also affected the phylogenetic distribution of the TaERF genes as shown in Supplementary Figure 4B. Similarly, in 26 TaRAV genes motifs number ranged from 6 to 13. The longest motif is motifs 1 and 2 (60 amino acids), and the shortest motif is 15 (five amino acids). The motifs 1, 2, 3, 4, and 5 are present in all the members of the TaRAV family genes. The motif 14 (15 amino acids long), present in three sites, is the least frequent. The TaRAV genes are clustered into three major clades, and the genes in close clades have similar motif compositions. The motif 15 (five amino acid long) shows specificity to one clade of the TaRAV family, which is not present in other TaRAV genes as shown in Supplementary Figure 4C.

In 66 TaAP2 genes, the number of motifs present per gene ranged from 4 to 10. The longest motif 11 (200 amino acid long) is present only in three sites and motifs 6, 12, and 13 are the shortest motifs present in 62, 34, and 17 sites. The motifs 5 (16 amino acids long) and 6 (12 amino acid long) are most frequent, present in all the members of TaAP2 family genes. The TaAP2 genes with similar motif patterns are clustered together in the same branch as adjacent leaves in the phylogenetic tree as shown in Supplementary Figure 4D. The details of the conserved motif discovered in the four TaAP2/ERF TF gene families are given in Supplementary Table 3.

As the genes with similar motif composition, gene structure and grouped closely in the phylogenetic tree may have similar functions, 351 genes were selected as representative of the whole TaAP2/ERF gene family based on the motif composition, gene structure, and phylogenetic proximity with at least 90% confidence, for further analysis.

### Genomic Distribution of TaAP2/ERF Genes in Wheat

**Figure 4:**
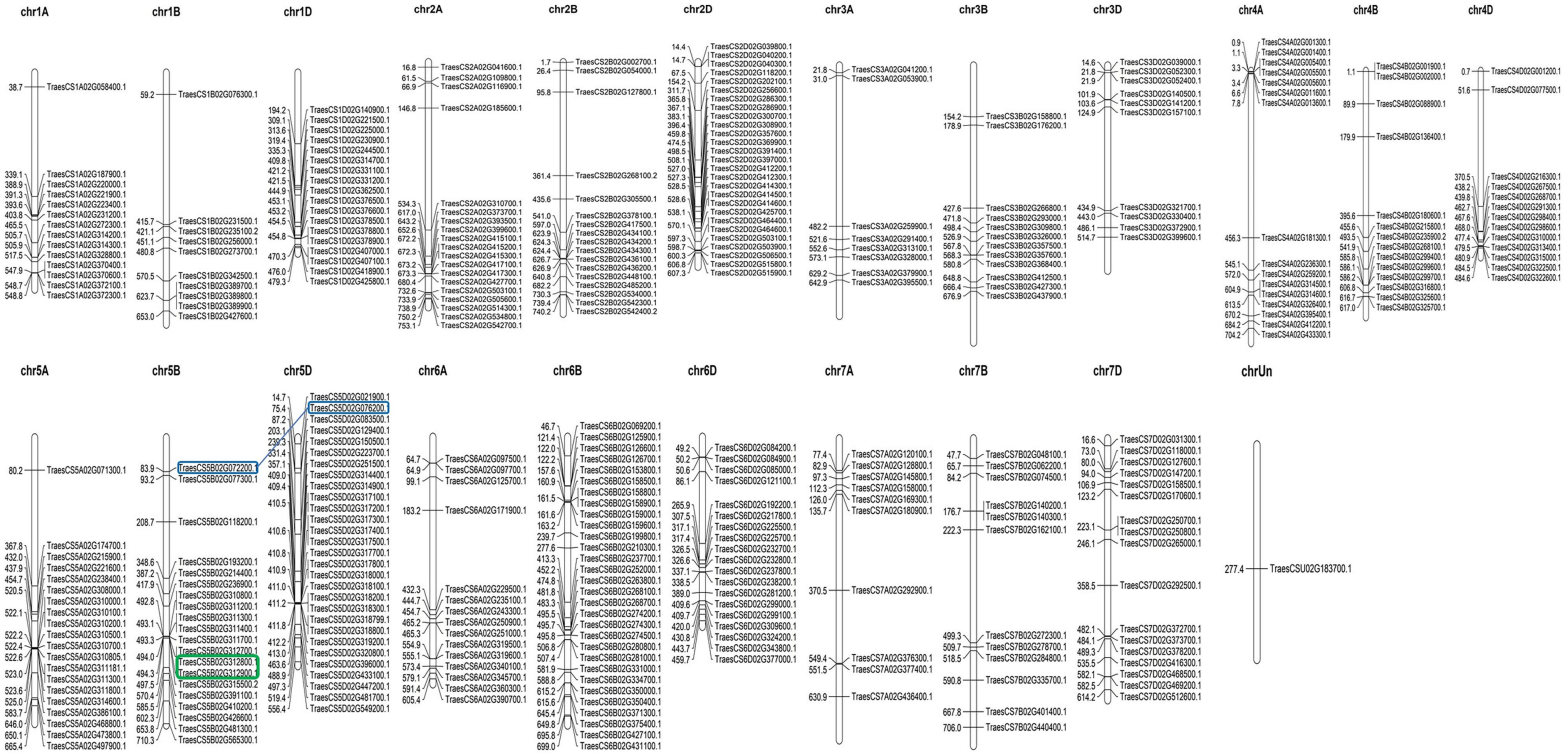
Distribution of 351 TaAP2/ERF TF genes in 21 wheat chromosomes and one unknown location generated in MapChart software. The relative positions of the genes on each chromosome are displayed at the top of each chromosome. The number of TaAP2/ERF TF genes per chromosome ranged from 30 in chromosome 6B to 1 as on the unknown position. Among the 128 pairs of segmental duplicates detected in TaAP2/ERF family TF genes, the one pair tandem duplicate is marked with a green box and one pair of segmental duplicated genes with the highest sequence similarity and identity of 99.18% is marked with blue boxes connected with a blue line. Gene duplication analysis was done based on the similarity and identity matrix of the full-length protein sequence generated from the Sequence Identity and Similarity (SIAS) online tool.

As a hexaploid, wheat contains three sub-genomes (A, B, and D). The selected 351 TaAP2/ERF genes were unevenly distributed in all the 21 chromosomes of the wheat genome. The number of TaAP2/ERF genes ranged from 30 in chromosome 6B, followed by 29 in chromosome 5D, while only one with unknown position, as visualized with MapChart (Figure 4). The genome-wide distribution of genes was 102 genes in sub-genome A, 115 genes in sub-genome B, 133 genes in D sub-genome, and one gene in unknown position.

Gene duplication widely contributes to the diversity and evolution of gene families. There are two types of gene duplication events: segmental duplication and tandem duplication. There were 128 pairs of segmental duplicated TaAP2/ERF genes, where one gene pair (*TraesCS5B02G312900.1* and *TraesCS5B02G312800.1*) was duplicated within 5B chromosome and located one after another, separated by 2.51-Kb distance between them, which was characterized as tandem duplicates. The highest sequence similarity and identity of 99.18% was present between segmental duplicated gene pair: *TraesCS5D02G076200.1* and *TraesCS5B02G072200.1*, present in chromosomes 5D and 5B, respectively, closely followed by the similarity of 99.13% and identity of 98.27% between gene pair *TraesCS1D02G244500.1* and *TraesCS1B02G256000.1*, present in chromosomes 1D and 1B, respectively (Figure 4; Supplementary Table 4). Can you dig deeper into A, B, and D genomes, such as how many on them separately, similarity and difference?

### *In silico* Expression Analysis and Analysis of the Cis-Regulatory Element of TaAP2/ERF Genes

The gene expression pattern is correlated with the biological function of a gene. The *in silico* expression pattern analysis of 351 TaAP2/ERF genes under abiotic stress using wheat expression database shows the genes are grouped into two major clusters. Most of the genes (91%) are grouped in a bigger cluster showing relatively lower expression with few genes showing moderately higher expression, and only 30 genes (9%) are grouped in a smaller cluster showing higher expression under abiotic stress (Supplementary Figure 5).

**Figure 5:**
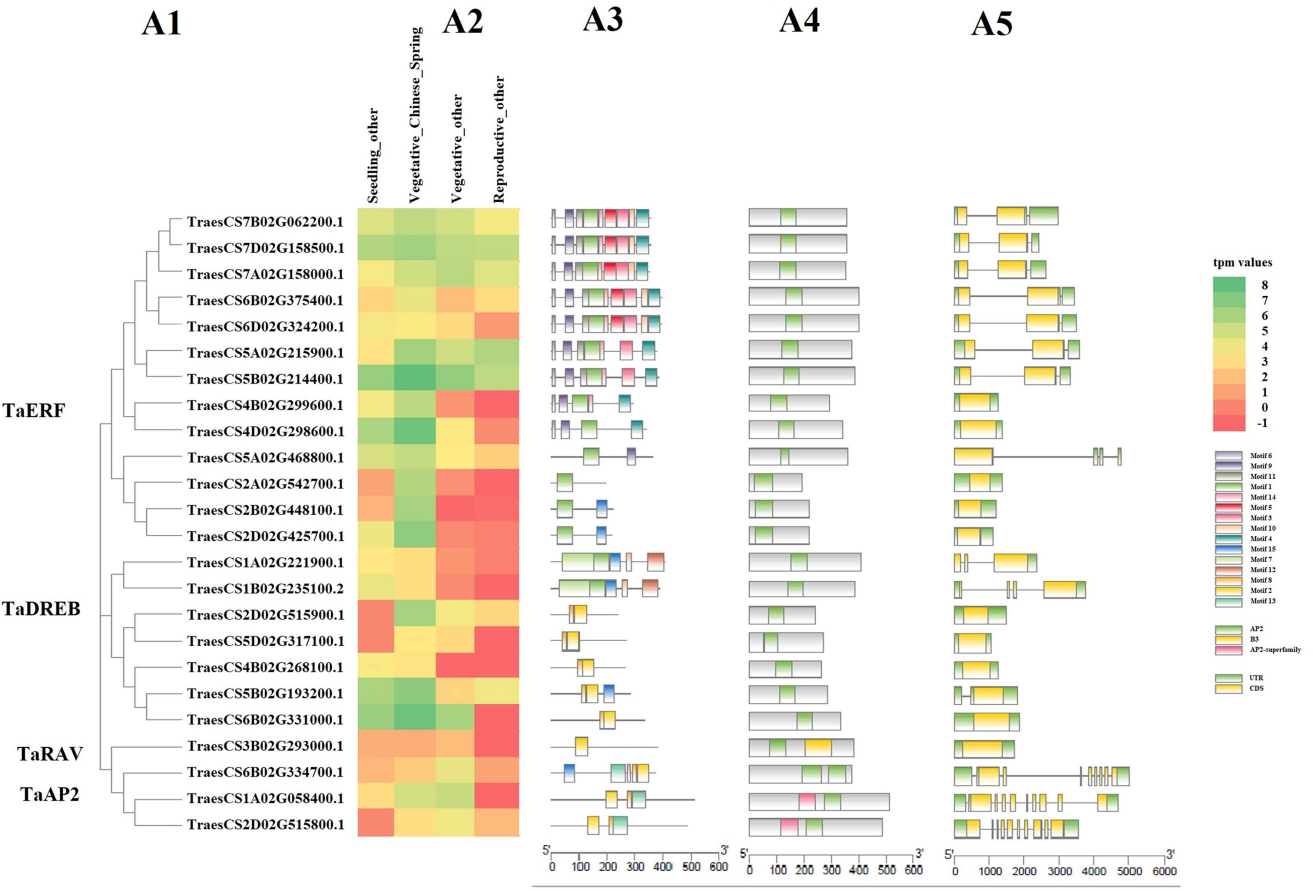
Heatmap showing the expression pattern of selected 24 TaAP2/ERF TF genes under abiotic stress using wheat expression transcriptome data **(A2)**, along with their conserved motif composition **(A3)**, protein domains **(A4)**, gene structure **(A5)**, and phylogenetic distribution **(A1)**. In the heatmap, the intensity of color shows the level of gene expression, where the red color shows the least expressed gene, and the green color shows the highest expressed gene. In the motif graph, the colored blocks represent the positions of motifs in corresponding proteins coding TF genes, block size indicates the length of motifs and grey lines connecting the colored bocks represent the non-conserved sequences. In the protein domain graph, the green blocks represent the AP2 domain, yellow blocks represent the B3 domain, and pink blocks represent the AP2 superfamily domain. In the gene structure graph, the yellow blocks are exons, green blocks are UTR, and grey lines are introns. The relative position of each motif, domain, and exon, intron, and UTR can be determined with the help of scale displayed just below the corresponding graph.

The expression profile of selected 24 TF genes with at least 1.4 average tpm value and representing all four gene families, along with their motif composition, protein domains, gene structure, and phylogenetic distribution is shown in Figure 5, which clearly shows that some genes are up-regulated, while some are down-regulated under HS in different genotypes.

Cis-regulatory element (CRE) analysis in 2 K promoter sequence of selected 24 TF genes in PlantCARE database revealed the presence of 2,992 CRE of 99 different types. These CRE can be broadly categorized into seven different groups as: (1) light-responsive elements (27 elements); (2) hormone-responsive elements (13 elements); (3) environmental stress-related elements (seven elements); (4) development-related elements (14 elements); (5) promoter-related elements (four elements); (6) site-binding-related elements (six elements); and (7) other elements (28 elements) with unknown function. Among these cis-acting elements identified in this study, CAAT-box and TATA-box (promoter related elements), STRE and Unnamed-4 (other elements) are present in all genes, followed by MYB and MYC (other elements), As-1 and CGTCA-motif (hormone-responsive elements) and ABRE (hormone-responsive related element) are present in >90% of genes. The most frequent CRE are: CAAT-box is recorded 609 times with maximum number (37 times) in *TraesCS2D02G515900.1*, TATA-box is recorded 358 times with maximum number (26 times) in *TraesCS7D02G158500.1*, and Unamed-4 is recorded 491 times with maximum number (38 times) in *TraesCS7D02G158500.1*, as shown in Supplementary Table 5.

### Morphology and Quantitative Gene Expression Analysis Using RT-qPCR

**Figure 6:**
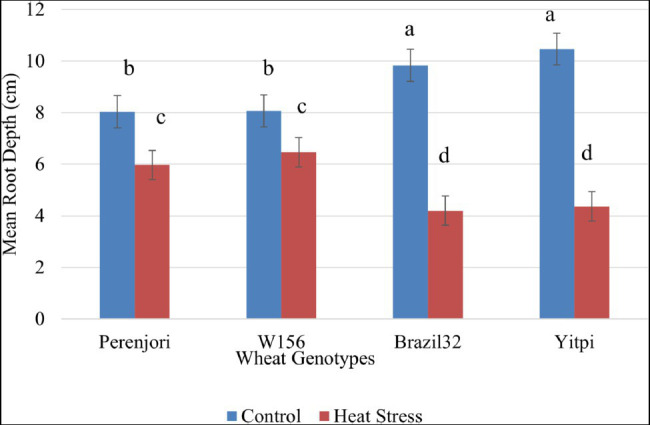
Effect of heat stress on average root depth of wheat genotypes at early seedlings stage. The bar chart shows variation in the mean root depth under control (blue color) and heat stress (orange color) condition, with an error bar on top of each bar. The means denoted by the same letter are not significantly different at *p* ≤ 0.05 by Tukey–Kramer HSD test.

The morphological assessment of 3 days heat-treated wheat seedlings shows a significant reduction in their root depth, both in tolerant and susceptible (Figure 6). The heat damage index showing damage due to HS was 35% higher in susceptible genotypes as compared to tolerant genotypes. The statistical analysis of the heat damage index shows a significant difference between groups with a value of *p* of 2.73E-06 at 95% confidence level.

**Figure 7:**
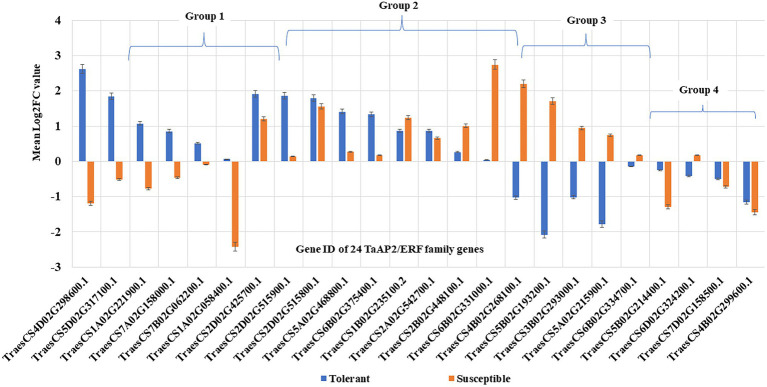
Gene regulation pattern of 24 TaAP2/ERF TF genes in tolerant and susceptible wheat genotypes based on RT-qPCR results. The 24 TF genes are classified into four groups according to the nature of their expression in different genotypes: **Group 1**. Genes up-regulated in tolerant but down-regulated in susceptible (six genes: *TraesCS4D02G298600.1*, *TraesCS5D02G317100.1*, *TraesCS1A02G221900.1*, *TraesCS7A02G158000.1*, *TraesCS7B02G062200.1*, and *TraesCS1A02G058400.1*); **Group 2**. Genes up-regulated in both tolerant and susceptible (nine genes: *TraesCS2D02G425700.1*, *TraesCS2D02G515900.1*, *TraesCS2D02G515800.1*, *TraesCS5A02G468800.1*, *TraesCS6B02G375400.1*, *TraesCS1B02G235100.2*, *TraesCS2A02G542700.1*, *TraesCS2B02G448100.1*, and *TraesCS6B02G331000.1*); **Group 3**. Genes up-regulated in susceptible but down-regulated in tolerant (five genes: *TraesCS4B02G268100.1*, *TraesCS5B02G193200.1*, *TraesCS3B02G293000.1*, *TraesCS5A02G215900.1*, and *TraesCS6B02G334700.1*); and **Group 4**. Genes down-regulated in both tolerant and susceptible (four genes: *TraesCS5B02G214400.1*, *TraesCS6D02G324200.1*, *TraesCS7D02G158500.1*, and *TraesCS4B02G299600.1*).

The RT-qPCR expression analysis of 24 TaAP2/ERF TF genes using gene-linked markers (Supplementary Table 6) in wheat seedlings showed differential expression of genes in tolerant and susceptible genotype (Figure 7). Some genes were up-regulated in both tolerant and susceptible genotypes, while some genes were down-regulated in both tolerant and susceptible genotypes, and some were regulated in opposite direction in tolerant and susceptible genotypes. The analysis of fold change (FC) values, calculated from the gene expression values, decided the significant gene expression difference between tolerant and susceptible genotypes. Eight TaAP2/ERF genes were significantly up-regulated and five were significantly down-regulated in tolerant genotype, whereas seven TaAP2/ERF genes were significantly up-regulated and four were significantly down-regulated in susceptible genotype (Supplementary Table 7). Further analysis of gene regulation pattern and classified them into four groups (Figure 7): Group 1 includes six genes (25%) up-regulated in tolerant genotypes but down-regulated in susceptible; Group 2 includes nine genes (37.50%) up-regulated in both tolerant and susceptible genotypes; Group 3 includes five genes (20.83%) up-regulated in susceptible ones but down-regulated in tolerant ones; and Group 4 include four genes (16.67%) down-regulated in both tolerant and susceptible ones.

## Discussion

Heat tolerance is a polygenic trait controlled by many genes and expression of the trait involves activation of molecular networks of TFs and heat-responsive genes (Singh et al., 2019). The AP2/ERF superfamily is one of the most diverse families of plant TFs, which are known for their regulatory role in biotic/abiotic stress-, growth-, and development-related gene expression (Debbarma et al., 2019). In this study, we identified 630 putative AP2/ERF superfamily genes in wheat through genome-wide homology search. The comprehensive characterization and expression analysis of TaAP2/ERF genes in heat-tolerant and heat-susceptible genotypes showed 18 differentially expressed genes (DEGs) among 24 genes expressed under HS. Among DEGs, eight were characterized as heat-tolerant genes, seven as heat-susceptible genes based on the specificity of expression patterns in contrasting genotypes, while two genes were significantly up-regulated, and one gene was significantly down-regulated in both tolerant and susceptible genotypes. The remaining six genes were not significantly regulated on either of the tolerant and susceptible genotypes.

### A Large Number of AP2/ERF Gene-Superfamily Genes Were Identified in Wheat

In this study, we identified 517 AP2/ERF genes with at least one AP2-domain in the wheat genome, which is four times higher than in jute (119 genes; Kabir et al., 2021), more than three times higher than in buckwheat (134 genes), *Arabidopsis* (147 genes), rice (164 genes), foxtail millet (171 genes), and oil palm (172 genes; Nakano et al., 2006; Lata et al., 2014; Liu et al., 2019; Zhou and Yarra, 2021) and more than double as in sweet potato (198 genes) and durum wheat (271; Faraji et al., 2020; He et al., 2021). This larger number of genes signifies the hexaploid nature of the wheat genome. This gene number is also way higher than the 322 AP2/ERF genes identified in wheat in a previous report (Riaz et al., 2021), which does not clearly address the AP2 and RAV family genes.

The protein domain analysis classified wheat AP2/ERF TF (TaAP2/ERF) genes into three families as TaERF (with single AP2 domain), TaAP2 (with two AP2 domains), and TaRAV (with single AP2 domain and single B3 domain). This is in line with the classification of the AP2/ERF family genes in *Arabidopsis* (Feng et al., 2020). The domain sequence analysis of TaERF family genes shows a highly conserved “WLG” motif in almost all members of TaERF family genes, which comply with the results of Liu S. et al. (2013) and Kabir et al. (2021). The clear differentiation in the presence of conserved amino acids in 14th and 19th positions along domain sequence classified them into three sub-families as TaDREB (169 genes), TaERF (238 genes), and others (18 genes; Supplementary Figures 1–3). Thus, classifying them into five sub-families of TaAP2/ERF superfamily TF genes: TaERF (238 genes), TaDREB (169 genes), TaAP2 (66 genes), TaRAV (26 genes), and others (18 genes). The classification is similar to the classification of Sakuma et al. (2002) and Nakano et al. (2006); however, the number of genes in each sub-family is much smaller in *Arabidopsis* such as 14 AP2 gene, six RAV genes, 56 DREB genes, 65 ERF genes, and four other sub-family genes. This expansion in gene numbers might be due to the polyploidy nature of the wheat genome with three sub-genomes. The direct effect of ploidy level on the amount of chromatin in the nucleus and copy number of each genes has been reported (Robinson et al., 2018). In this study, four additional TaAP2 genes (*TraesCS6B02G158500.1*, *TraesCS6B02G158800.1*, *TraesCS6B02G158900.1*, and *TraesCS6B02G159000.1*) were identified as compared to 62 TaAP2 genes reported previously by Zhao et al. (2019). In addition, one TaRAV family gene, 62 TaAP2 family genes, 112 TaERF sub-family genes, and 77 TaDREB sub-family genes were unique as compared to the TaAP2/ERF genes reported by Riaz et al. (2021).

The higher number of genes in the TaERF and TaDREB sub-family is also associated with the higher number of intronless genes as compared to TaAP2 family genes. Gene structure analysis shows that most of the genes (>73%) in TaERF and (>80%) in the TaDREB sub-family are intronless, while only four (0.06%) TaAP2 genes are intronless (Supplementary Figures 4A–D). Also, among 351 TaAP2/ERF genes, 127 pairs of segmental duplicates (four pairs from TaRAV family, 11 pairs from TaAP2 family, 53 pairs from TaDREB sub-family, and 59 pairs from the TaERF family) with a single pair of a tandem duplicate from TaDREB sub-family genes were identified. The higher number of duplicated genes in the TaERF and TaDREB sub-family as compared to the TaAP2 family is due to the presence of intronless genes in these families, which induce rapid expansion of gene family through gene duplication. The expansion and maintenance of gene families arise from gene duplication events, where tandem duplication makes gene clusters and segmental duplication generates homologous genes, thus increasing the number of genes in gene family (Cannon et al., 2004). In addition, detection of a larger number of segmental duplicated genes indicates the presence of homologous genes contributing to the expansion of TaAP2/ERF gene families, mainly from TaERF and TaDREB sub-family genes. This finding is consistent with the 250 segmental duplicates and 35 tandem duplicate genes reported in the AP2/ERF family of *Brassica rapa*, with ERF genes dominant among duplicates (Liu Z. et al., 2013). The comparable number of duplicated genes in hexaploid wheat genome and diploid *B. rapa* genome might be due to the several rounds of whole-genome duplication events that occurred in the Brassica genome significantly increasing the number of duplicated genes (Cannon et al., 2004), whereas the relatively smaller number of segmental duplicated AP2/ERF genes was reported in maize (21 pairs; Hao et al., 2020) and dark jute (eight pairs; Kabir et al., 2021). This is explained by the diploid genome of maize and dark jute with 10 and 14 chromosomes, respectively, as compared to the hexaploid genome with three copies of sub-genome and 21 chromosomes in wheat.

### Expression of TaAP2/ERF Genes Under Heat Stress and Its Implication

A systematic gene expression analysis of TaAP2/ERF genes under abiotic stress, using transcription data from the wheat expression database, shows differential expression of genes, with a major cluster showing moderately and less expressed genes and small clusters (30 genes) showing relatively highly expressed genes (Supplementary Figure 5). This shows that some TaAP2/ERF genes are more important than others in response to abiotic stresses. The regulatory role of AP2/ERF family genes under various abiotic stress has also been reported in a diverse range of plant species (Faraji et al., 2020; Hao et al., 2020; He et al., 2021; Kabir et al., 2021; Riaz et al., 2021; Xing et al., 2021; Zhou and Yarra, 2021).

Gene expression data are an important parameter to elucidate the function of a gene under given stress and in a specific genotype. The qRT-PCR gene expression analysis of 24 TaAP2/ERF genes in four wheat genotypes under HS condition shows a prominent effect of genotypes on gene expression. Eight genes were significantly up-regulated, and five genes were significantly down-regulated in tolerant genotypes, whereas seven genes were significantly up-regulated, and four genes were significantly down-regulated in susceptible genotypes (Table 1). Expression of AP2/ERF family genes under HS has also been reported in maize (Mohamed et al., 2019), *Arabidopsis* (Niu et al., 2020), *Solanum lycopercicum* (Deng et al., 2020), and rice (Matsukura et al., 2010). The increased expression of TaAP2/ERF genes under HS was also reported in wheat cv. Chinese spring (Riaz et al., 2021). In this study, three genes (*TraesCS4B02G268100.1*, *TraesCS5B02G193200.1*, and *TraesCS6B02G331000.1*) were significantly up-regulated in susceptible genotypes and two genes (*TraesCS4B02G268100.1* and *TraesCS5B02G193200.1*) were significantly down-regulated in tolerant genotypes that are also reported as up-regulated genes under HS in Chinese Spring (Riaz et al., 2021). Chinese spring was reported as heat-susceptible wheat genotype by Qin et al. (2008). In addition to these three genes commonly expressed in Chinese spring and susceptible genotype in this study, we have identified two genes (*TraesCS1B02G235100.2* and *TraesCS2B02G448100.1*) significantly up-regulated in susceptible genotypes and two genes (*TraesCS3B02G293000.1* and *TraesCS5A02G215900.*1) significantly down-regulated in tolerant genotypes, thus contributing to heat susceptibility. The genes expressed similarly in susceptible genotypes in this study and in Chinese Spring show significant consistency in their expression in different genotypic backgrounds and provide strong evidence that these genes can be used for molecular screening of the susceptible wheat genotypes on large scale. The additional four genes associated with heat susceptibility in this study might be genotype-specific susceptible genes.

**Table 1:** Gene expression pattern of candidate TaAP2/ERF genes in tolerant and susceptible genotypes after 3 days heat treatment at 35 ± 1°C, normalized with β-actin as standard and control at 25 ± 1°C, along with their associated gene family and traits.

Gene ID	Mean FC values		Mean Log_2_FC values		Gene family	Trait
	Tolerant	Susceptible	Tolerant	Susceptible		
*TraesCS4D02G298600*	6.29	0.51	**2.62**	**−1.20**	TaERF	Highly heat-tolerant gene
*TraesCS5D02G317100*	5.94	0.74	**1.84**	−0.52	TaDREB	Moderately highly heat-tolerant gene
*TraesCS1A02G221900*	2.11	0.58	**1.08**	−0.78	TaDREB	Moderately highly heat-tolerant gene
*TraesCS2D02G515900*	4.03	1.21	**1.86**	0.15	TaDREB	Heat tolerant
*TraesCS5A02G468800*	3.81	1.23	**1.41**	0.27	TaERF	Heat tolerant
*TraesCS6B02G375400*	4.34	1.27	**1.34**	0.18	TaERF	Heat tolerant
*TraesCS1A02G058400*	1.05	0.22	0.07	**−2.42**	TaAP2	Heat tolerant
*TraesCS5B02G214400*	1.09	0.42	−0.25	**−1.29**	TaERF	Heat tolerant
*TraesCS4B02G268100*	0.65	4.60	**−1.02**	**2.20**	TaDREB	Highly heat-susceptible gene
*TraesCS5B02G193200*	0.44	3.33	**−2.08**	**1.71**	TaDREB	Highly heat-susceptible gene
*TraesCS3B02G293000*	0.50	2.18	**−1.02**	0.94	TaRAV	Moderately highly heat-susceptible gene
*TraesCS5A02G215900*	0.52	5.44	**−1.79**	0.74	TaERF	Moderately highly heat-susceptible gene
*TraesCS6B02G331000*	1.69	6.72	0.04	**2.74**	TaDREB	Heat-susceptible gene
*TraesCS1B02G235100*	1.83	2.36	0.86	**1.23**	TaDREB	Heat-susceptible gene
*TraesCS2B02G448100*	1.61	2.86	0.26	**1.01**	TaERF	Heat-susceptible gene
*TraesCS2D02G425700*	3.93	2.51	**1.91**	**1.20**	TaERF	Neutral gene
*TraesCS2D02G515800*	9.98	3.17	**1.79**	**1.56**	TaAP2	Neutral gene
*TraesCS4B02G299600*	0.54	0.41	**−1.15**	**−1.45**	TaERF	Neutral gene
Number of significantly up-regulated			8	7		
Number of significantly down-regulated			5	4		
Total number of significantly regulated genes			13	11		

Bold fonts represent significantly up-regulated genes (positive value) and significantly down-regulated (negative value) genes.

At the same time, five genes (*TraesCS4D02G298600.1*, *Traes CS5D02G317100.1*, *TraesCS2D02G515900.1*, *TraesCS5A02G46 8800.1*, and *TraesCS6B02G375400.1*) are significantly up-regulated in tolerant genotypes and two genes (*TraesCS1A02G058400.1* and *TraesCS5B02G214400.1*) are significantly down-regulated in susceptible genotypes, which are considered to confer heat tolerance in wheat. In addition, one gene (*TraesCS1A02G221900.1*) which was significantly up-regulated in heat-tolerant genotype was also reported to enhance tolerance to heat, drought, and salinity stress in transgenic *Arabidopsis* (Niu et al., 2020). This gene is associated with heat tolerance in wheat. This also reveals that the TaAP2/ERF genes have the ability to co-express under multiple stress conditions, which might be due to the presence of multiple cis-regulatory induced by abiotic stress in their promoter region. The stress-responsive gene expression is regulated by cis-regulatory elements (CRE) present in the promoter region and the variation in the cis-regulatory elements in a gene results in variation in their regulatory role, stress response, and expression pattern (Ramírez-González et al., 2018; Riaz et al., 2021). The CRE, including CAAT-box, TATA-box, MYB, MYC, CGTCA-motif, As-1, and ABRE, is identified in the promoter sequence of almost all heat-induced TaAP2/ERF genes, which shows their association with heat response. The dominance of ABRE and ABRE-related elements (ABRE3a, ABRE4, AT-ABRE) in the TaAP2/ERF promoter sequence indicated they are highly responsive to HS. The ABRE has also been reported as abiotic stress-responsive elements in rice (Lenka and Bansal, 2019), barley (Guo et al., 2016), maize (Hao et al., 2021), and durum wheat (Yamaguchi-Shinozaki and Shinozaki, 2006). In addition, the CREs such as dehydration-responsive element (DRE and DRE-core), gibberellin-responsive element (GARE-motif), auxin-responsiveness elements (AuxRR-core), drought-inducibility element (MBS), light-responsive element (G-box and TCT-motif), MYC and MYB elements identified in this study have also been reported as TF genes responsible for the expression of abiotic stress-responsive genes in different plant species (Feng et al., 2020). This strongly suggests that the presence of abiotic stress-responsive CRE in the promoter region of TaAP2/ERF genes is responsible for the expression of these genes in wheat under HS conditions and co-express under multiple abiotic stresses.

Further, gene expression pattern analysis shows that six genes are significantly up-regulated in tolerant and significantly down-regulated or nonsignificantly up-regulated in susceptible genotypes; five genes are significantly up-regulated in susceptible and significantly down-regulated or nonsignificantly up-regulated in tolerant genotypes. These genes are very specific in their expression pattern in tolerant and susceptible genotypes, which might be useful in differentiating contrasting genotypes. Two genes are significantly up-regulated in both tolerant and susceptible genotypes, and one gene is significantly down-regulated in both tolerant and susceptible genotypes, indicating these genes are universal to heat responses that might be less useful in the selection process despite significant expression. Based on the extent of their expressions, the heat-tolerant genes (eight genes) are categorized as highly heat tolerant, moderately highly heat tolerant, and heat tolerant, and heat-susceptible genes (seven genes) are categorized as highly heat susceptible, moderately highly heat susceptible and heat susceptible (Table 1).

Thus, we identified eight heat-tolerant genes and seven heat-susceptible genes in TaAP2/ERF superfamily, consisting of TaERF, TaDREB, and TaAP2 genes in the tolerant category and TaERF, TaDREB, and TaRAV genes in the susceptible category. This reveals that the ERF and DREB sub-family genes are mostly expressed both in tolerant and susceptible genotypes, whereas some AP2 genes are expressed only in heat tolerant, and some RAV genes are expressed only in heat-susceptible genotypes. The higher expression of TaERF and TaDREB sub-family genes might be associated with the family-specific conserve motifs, which are responsible for maintaining the family-specific functions. It is also supported by the presence of a larger number of intronless genes in these sub-families, leading to functional evolution and stress adaptation (Shu et al., 2015; Faraji et al., 2020). Similar nature of AP2/ERF gene structure has also been reported in *Arabidopsis*, tartary buckwheat (Lata et al., 2014), sweet potato (He et al., 2021), dark jute (Kabir et al., 2021). Thus, structural features of genes are directly associated with the expression pattern of genes, which determines their functional characters and makes TaERF and TaDREB sub-family genes more heat-responsive as compared to TaAP2 and TaRAV family genes under HS. The heat-responsive genes identified in this study can be utilized as a molecular marker for mass screening of wheat genotypes, gene introgressing, and pyramiding of multiple heat-tolerant genes in the common background to develop wheat resilient genotypes with multiple heat-tolerant genes. The heat tolerance conferred by multiple tolerance gene with additive effect is considered more stable as compared to the heat tolerance due to a single tolerant gene.

## Conclusion

This study identified, classified, and characterized 517 AP2/ERF TF genes in wheat. The genes in four sub-families of the TaAP2/ERF superfamily show variation in conserved motif and gene structures, which affect the gene duplication, leading to variation in gene number and response to HS. The comparative gene expression analysis in contrasting genotypes reveals candidate genes that have specific expression patterns in tolerant and susceptible genotypes, which provides strong evidence that genes up-regulated in tolerant and down-regulated in susceptible genotypes can be considered as heat-tolerant genes and the genes up-regulated in susceptible and down-regulated in tolerant genotypes are heat-susceptible genes. Thus, genes with expression patterns contrasting in tolerant and susceptible genotypes are especially valuable for heat tolerance breeding in wheat. Further detailed analysis and validation of these genes at the reproductive stage may be required to ensure that these genes provide complete heat tolerance in wheat under HS.

## Data Availability Statement

The datasets presented in this study can be found in online repositories. The names of the repository/repositories and accession number(s) can be found in the article/Supplementary Material.

## Author Contributions

MM did conceptualization, investigation, methodology, data curation, formal analysis, and writing—original draft. HL was involved in conceptualization, funding acquisition, methodology, formal analysis, resources, supervision, and writing—review and editing. GY provided conceptualization, funding acquisition, methodology, formal analysis, resources, supervision, and writing—review and editing. All authors contributed to the article and approved the submitted version.

## Funding

This research was funded by the University of Western Australia International Fee Scholarship and a University Postgraduate Award, as a sponsorship to PhD of the MM. The research is partly supported by Global Innovation Linkages Project (GIL53853) from the Australian Department of Industry, Science, Energy and Resources.

## Conflict of Interest

The authors declare that the research was conducted in the absence of any commercial or financial relationships that could be construed as a potential conflict of interest.

## Publisher’s Note

All claims expressed in this article are solely those of the authors and do not necessarily represent those of their affiliated organizations, or those of the publisher, the editors and the reviewers. Any product that may be evaluated in this article, or claim that may be made by its manufacturer, is not guaranteed or endorsed by the publisher.

## Footnotes

^1^ https://wheat-urgi.versailles.inra.fr/Seq-Repository/Assemblies

^2^ http://pfam.xfam.org/family/PF00847

^3^ http://hmmer.org/

^4^ https://www.ncbi.nlm.nih.gov/Structure/bwrpsb/bwrpsb.cgi

^5^ http://web.expasy.org/protparam/

^6^ https://www.geneious.com/prime/

^7^ https://itol.embl.de/tree

^8^ https://meme-suite.org/meme/tools/meme

^9^ http://gsds.gao-lab.org/

^10^ https://github.com/CJ-Chen/TBtools

^11^ https://urgi.versailles.inra.fr/jbrowseiwgsc/gmod_jbrowse/

^12^ https://www.wur.nl/en/Research-Results/Research-Institutes/plant-research/Biometris-1/Software-Service/Download-MapChart.htm

^13^ http://imed.med.ucm.es/Tools/sias.html

^14^ http://www.wheat-expression.com/

^15^ http://bioinformatics.psb.ugent.be/webtools/plantcare/html/

^16^ https://urgi.versailles.inra.fr/jbrowseiwgsc/gmod_jbrowse/

^17^ https://www.geneious.com/prime/

## References

[ref1] Ali S. Rizwan M. Arif M. S. Ahmad R. Hasanuzzaman M. Ali B.. (2020). Approaches in enhancing thermotolerance in plants: an updated review. J. Plant Growth Regul. 39, 456–480. doi: 10.1007/s00344-019-09994-x

[ref2] Alonso J. M. Stepanova A. N. Leisse T. J. Kim C. J. Chen H. Shinn P.. (2003). Genome-wide Insertional mutagenesis of *Arabidopsis thaliana*. Science 301, 653–657. doi: 10.1126/science.1086391, PMID: 12893945

[ref3] Alsamadany H. (2016). Diversity and Genetic Studies of Heat Tolerance in Wheat. PhD thesis. The University of Western Australia.

[ref4] Appels R. Eversole K. Feuillet C. Keller B. Rogers J. Stein N.. (2018). Shifting the limits in wheat research and breeding using a fully annotated reference genome. Science 361:eaar7191. doi: 10.1126/science.aar7191, PMID: 30115783

[ref5] Begcy K. Nosenko T. Zhou L. Z. Fragner L. Weckwerth W. Dresselhaus T. (2019). Male sterility in maize after transient heat stress during the tetrad stage of pollen development. Plant Physiol. 181, 683–700. doi: 10.1104/pp.19.00707, PMID: 31378720 PMC6776839

[ref6] Cannon S. B. Mitra A. Baumgarten A. Young N. D. May G. (2004). The roles of segmental and tandem gene duplication in the evolution of large gene families in *Arabidopsis thaliana*. BMC Plant Biol. 4:10. doi: 10.1186/1471-2229-4-10, PMID: 15171794 PMC446195

[ref7] Chen H. Je J. Song C. Hwang J. E. Lim C. O. (2012). A proximal promoter region of *Arabidopsis* DREB2C confers tissue-specific expression under heat stress. J. Integr. Plant Biol. 54, 640–651. doi: 10.1111/j.1744-7909.2012.01137.x, PMID: 22716647

[ref8] Debbarma J. Sarki Y. N. Saikia B. Boruah H. P. D. Singha D. L. Chikkaputtaiah C. (2019). Ethylene response factor (ERF) family proteins in abiotic stresses and CRISPR–Cas9 genome editing of ERFs for multiple abiotic stress tolerance in crop plants: a review. Mol. Biotechnol. 61, 153–172. doi: 10.1007/s12033-018-0144-x, PMID: 30600447

[ref9] Deng M. H. Lv J. H. Wang Z. R. Zhu H. S. Yang Z. A. Yue Y. L.. (2020). Two promoter regions confer heat-induced activation of SlDREBA4 in *Solanum lycopersicum*. Biochem. Biophys. Res. Commun. 524, 689–695. doi: 10.1016/j.bbrc.2020.01.153, PMID: 32033747

[ref10] Driedonks N. Rieu I. Vriezen W. H. (2016). Breeding for plant heat tolerance at vegetative and reproductive stages. Plant Reprod. 29, 67–79. doi: 10.1007/s00497-016-0275-9, PMID: 26874710 PMC4909801

[ref11] El-Gebali S. Mistry J. Bateman A. Eddy S. R. Luciani A. Potter S. C.. (2019). The Pfam protein families database in 2019. Nucleic Acids Res. 47, D427–D432. doi: 10.1093/nar/gky995, PMID: 30357350 PMC6324024

[ref12] Erdayani E. Nagarajan R. Grant N. P. Gill K. S. (2020). Genome-wide analysis of the HSP101/CLPB gene family for heat tolerance in hexaploid wheat. Sci. Rep. 10:3948. doi: 10.1038/s41598-020-60673-4, PMID: 32127546 PMC7054433

[ref13] Fang Z. Jiang W. He Y. Ma D. Liu Y. Wang S.. (2020). Genome-wide identification, structure characterization, and expression profiling of Dof transcription factor gene family in wheat (*Triticum aestivum* L.). Agronomy 10:294. doi: 10.3390/agronomy10020294

[ref14] FAO IFAD UNICEF WEP WHO (2019). “The state of food security and nutrition in the world 2019,” in Safeguarding against Economic Slowdowns and Downturns. ed. Holleman C. (Rome: Food and Agriculture Organization of the United Nations).

[ref15] Faraji S. Filiz E. Kazemitabar S. K. Vannozzi A. Palumbo F. Barcaccia G.. (2020). The AP2/ERF gene family in *Triticum durum*: genome-wide identification and expression analysis under drought and salinity stresses. Gene 11, 1464–1488. doi: 10.3390/genes11121464, PMID: 33297327 PMC7762271

[ref16] Feng K. Hou X. L. Xing G. M. Liu J. X. Duan A. Q. Xu Z. S.. (2020). Advances in AP2/ERF super-family transcription factors in plant. Crit. Rev. Biotechnol. 40, 750–776. doi: 10.1080/07388551.2020.1768509, PMID: 32522044

[ref17] Gu L. Jiang T. Zhang C. Li X. Wang C. Zhang Y.. (2019). Maize HSFA2 and HSBP2 antagonistically modulate raffinose biosynthesis and heat tolerance in *Arabidopsis*. Plant J. 100, 128–142. doi: 10.1111/tpj.14434, PMID: 31180156

[ref18] Guerin C. Roche J. Allard V. Ravel C. Mouzeyar S. Bouzidi M. F. (2019). Genome-wide analysis, expansion and expression of the NAC family under drought and heat stresses in bread wheat (*T. aestivum* L.). PLoS One 14:e0213390. doi: 10.1371/journal.pone.0213390, PMID: 30840709 PMC6402696

[ref19] Guo B. Wei Y. Xu R. Shen L. Luan H. Lv C.. (2016). Genome-wide analysis of APETALA2/ethylene-responsive factor (AP2/ERF) gene family in barley (*Hordeum vulgare* L.). PLoS One 11:e0161322. doi: 10.1371/journal.pone.0161322, PMID: 27598245 PMC5012588

[ref20] Hall B. G. (2013). Building phylogenetic trees from molecular data with MEGA. Mol. Biol. Evol. 30, 1229–1235. doi: 10.1093/molbev/mst012, PMID: 23486614

[ref21] Hao L. Shi S. Guo H. Li M. Hu P. Wei Y.. (2020). Genome-wide identification and expression profiles of ERF subfamily transcription factors in *Zea mays*. PeerJ 8:e9551. doi: 10.7717/peerj.9551, PMID: 32742811 PMC7370932

[ref22] Hao L. Shi S. Guo H. Zhang J. Li P. Feng Y. (2021). Transcriptome analysis reveals differentially expressed MYB transcription factors associated with silicon response in wheat. Sci. Rep. 11:4330. doi: 10.1038/s41598-021-83912-8, PMID: 33619339 PMC7900239

[ref23] He S. Hao X. He S. Hao X. Chen X. (2021). Genome-wide identification, phylogeny and expression analysis of AP2/ERF transcription factors family in sweet potato. BMC Genomics 22:748. doi: 10.1186/s12864-021-08043-w, PMID: 34656106 PMC8520649

[ref24] Hong B. Ma C. Yang Y. Wang T. Yamaguchi-Shinozaki K. Gao J. (2009). Over-expression of AtDREB1A in chrysanthemum enhances tolerance to heat stress. Plant Mol. Biol. 70, 231–240. doi: 10.1007/s11103-009-9468-z, PMID: 19234675

[ref25] Hu Y. X. Wang Y. H. Liu X. F. Li J. Y. (2004). *Arabidopsis* RAV1 is down-regulated by brassinosteroid and may act as a negative regulator during plant development. Cell Res. 14, 8–15. doi: 10.1038/sj.cr.7290197, PMID: 15040885

[ref26] Jofuku K. D. den Boer B. G. Van Montagu M. Okamuro J. K. (1994). Control of *Arabidopsis* flower and seed development by the homeotic gene APETALA2. Am. Soc. Plant Physiol. 6, 1211–1225. 10.1105/tpc.6.9.1211 PMC160514 7919989

[ref27] Kabir S. M. Sabbir T. M. Hossain K. K. Bashar U. H. Ahmed B. Emdad E. M.. (2021). Genome-wide identification and expression profiling of AP2/ERF superfamily genes under stress conditions in dark jute (*Corchorus olitorius* L.). Ind. Crop. Prod. 166:113469. doi: 10.1016/j.indcrop.2021.113469

[ref28] Kagaya Y. Ohmiya K. Hattori T. (1999). RAV1, a novel DNA-binding protein, binds to bipartite recognition sequence through two distinct DNA-binding domains uniquely found in higher plants. Nucleic Acids Res. 27, 470–478. doi: 10.1093/nar/27.2.470, PMID: 9862967 PMC148202

[ref29] Lata C. Mishra A. K. Muthamilarasan M. Bonthala V. S. Khan Y. Prasad M. (2014). Genome-wide investigation and expression profiling of AP2/ERF transcription factor superfamily in foxtail millet (*Setaria italica* L.). PLoS One 9:e113092. doi: 10.1371/journal.pone.0113092, PMID: 25409524 PMC4237383

[ref30] Lenka S. Bansal K. C. (2019). Abiotic stress responsive cis-regulatory elements (CREs) in rice (*Oryza sativa* L.) and other plants. [Preprint]. doi:10.31219/osf.io/n98t5

[ref31] Li J. Liu H. Yang C. Wang J. Yan G. Si P.. (2020). Genome-wide identification of MYB genes and expression analysis under different biotic and abiotic stresses in *Helianthus annuus* L. Ind. Crop. Prod. 143, 111924–111937. doi: 10.1016/j.indcrop.2019.111924

[ref32] Liu H. Able A. J. Able J. A. (2020). Transgenerational effects of water-deficit and heat stress on germination and seedling vigour-new insights from durum wheat microRNAs. Plants 9, 189–209. doi: 10.3390/plants9020189, PMID: 32033017 PMC7076468

[ref33] Liu Z. Kong L. Zhang M. Lv Y. Liu Y. Zou M.. (2013). Genome-wide identification, phylogeny, evolution and expression patterns of AP2/ERF genes and cytokinin response factors in *Brassica rapa* ssp. pekinensis. PLoS One 8:e83444. doi: 10.1371/journal.pone.0083444, PMID: 24386201 PMC3875448

[ref34] Liu M. Sun W. Ma Z. Zheng T. Huang L. Wu Q.. (2019). Genome-wide investigation of the AP2/ERF gene family in tartary buckwheat (*Fagopyum Tataricum*). BMC Plant Biol. 19:84. doi: 10.1186/s12870-019-1681-6, PMID: 30786863 PMC6381666

[ref35] Liu S. Wang X. Wang H. Xin H. Yang X. Yan J.. (2013). Genome-wide analysis of ZmDREB genes and their association with natural variation in drought tolerance at seedling stage of *Zea mays* L. PLoS Genet. 9:e1003790. doi: 10.1371/journal.pgen.1003790, PMID: 24086146 PMC3784558

[ref36] Liu G. Zha Z. Cai H. Qin D. Jia H. Liu C.. (2020). Dynamic Transcriptome analysis of anther response to heat stress during anthesis in thermotolerant rice (*Oryza sativa* L.). Int. J. Mol. Sci. 21, 1155–1172. doi: 10.3390/ijms21031155, PMID: 32050518 PMC7037497

[ref37] Livak K. J. Schmittgen T. D. (2001). Analysis of relative gene expression data using real-time quantitative PCR and the 2(-delta delta C(T)) method. Methods 25, 402–408. doi: 10.1006/meth.2001.1262 11846609

[ref38] Matsukura S. Mizoi J. Yoshida T. Todaka D. Ito Y. Maruyama K.. (2010). Comprehensive analysis of rice DREB2-type genes that encode transcription factors involved in the expression of abiotic stress-responsive genes. Mol. Gen. Genomics. 283, 185–196. doi: 10.1007/s00438-009-0506-y, PMID: 20049613

[ref39] Mohamed H. I. Ashry N. A. Ghonaim M. M. (2019). Physiological and biochemical effects of heat shock stress and determination of molecular markers related to heat tolerance in maize hybrids. Gesunde Pflanz. 71, 213–222. doi: 10.1007/s10343-019-00467-5

[ref40] Najafi S. Sorkheh K. Nasernakhaei F. (2018). Characterization of the APETALA2/ethylene-responsive factor (AP2/ERF) transcription factor family in sunflower. Sci. Rep. 8:11576. doi: 10.1038/s41598-018-29526-z, PMID: 30068961 PMC6070487

[ref41] Nakano T. Suzuki K. Fujimura T. Shinshi H. (2006). Genome-wide analysis of the ERF gene family in *Arabidopsis* and rice. Plant Physiol. 140, 411–432. doi: 10.1104/pp.105.073783, PMID: 16407444 PMC1361313

[ref42] NCEI NOAA National Centers for Environmental Information (2021). State of the Climate: Global Climate Report for Annual 2020. Available at: https://www.ncdc.noaa.gov/sotc/global/202013 (Accessed March 10, 2022).

[ref43] Niu X. Luo T. Zhao H. Su Y. Ji W. Li H. (2020). Identification of wheat DREB genes and functional characterization of TaDREB3 in response to abiotic stresses. Gene 740:144514. doi: 10.1016/j.gene.2020.144514, PMID: 32112985

[ref44] Ohme-Takagi M. Shinshi H. (1995). Ethylene-inducible DNA binding proteins that interact with an ethylene-responsive element. Plant Cell 7, 173–182. doi: 10.1105/tpc.7.2.173, PMID: 7756828 PMC160773

[ref45] Qian Y. Ren Q. Zhang J. Chen L. (2019). Transcriptomic analysis of the maize (*Zea mays* L.) inbred line B73 response to heat stress at the seedling stage. Gene 692, 68–78. doi: 10.1016/j.gene.2018.12.062, PMID: 30641208

[ref46] Qin D. Wu H. Peng H. Yao Y. Ni Z. Li Z.. (2008). Heat stress-responsive transcriptome analysis in heat susceptible and tolerant wheat (*Triticum aestivum* L.) by using wheat genome Array. BMC Genomics 9:432. doi: 10.1186/1471-2164-9-432, PMID: 18808683 PMC2614437

[ref47] Ramírez-González R. H. Borrill P. Lang D. Harrington S. A. Brinton J. Venturini L.. (2018). The transcriptional landscape of polyploid wheat. Science 361, 662–675. doi: 10.1126/science.aar6089, PMID: 30115782

[ref48] Riaz M. W. Lu J. Shah L. Yang L. Chen C. Mei X. D.. (2021). Expansion and molecular characterization of AP2/ERF gene family in wheat (*Triticum aestivum* L.). Front. Genet. 12:632155. doi: 10.3389/fgene.2021.632155, PMID: 33868370 PMC8044323

[ref49] Robinson D. O. Coate J. E. Singh A. Hong L. Bush M. Doyle J. J.. (2018). Ploidy and size at multiple scales in the *Arabidopsis* sepal. Plant Cell 30, 2308–2329. doi: 10.1105/tpc.18.00344, PMID: 30143539 PMC6241276

[ref50] Sakuma Y. Liu Q. Dubouzet J. G. Abe H. Shinozaki K. Yamaguchi-Shinozaki K. (2002). DNA-binding specificity of the ERF/AP2 domain of *Arabidopsis* DREBs, transcription factors involved in dehydration- and cold-inducible gene expression. Biochem. Biophys. Res. Commun. 290, 998–1009. doi: 10.1006/bbrc.2001.6299, PMID: 11798174

[ref51] Schilling S. Kennedy A. Pan S. Jermiin L. S. Melzer R. (2020). Genome-wide analysis of MIKC-type MADS-box genes in wheat: pervasive duplications, functional conservation and putative neofunctionalization. New Phytol. 225, 511–529. doi: 10.1111/nph.16122, PMID: 31418861

[ref52] Shrestha K. Pant S. Huang Y. (2021). Genome-wide identification and classification of lipoxygenase gene family and their roles in sorghum-aphid interaction. Plant Mol. Biol. 105, 527–541. doi: 10.1007/s11103-020-01107-7, PMID: 33387173

[ref53] Shu Y. Liu Y. Zhang J. Song L. Guo C. (2015). Genome-wide analysis of the AP2/ERF superfamily genes and their responses to abiotic stress in *Medicago truncatula*. Front. Plant Sci. 6:1247. doi: 10.3389/fpls.2015.01247, PMID: 26834762 PMC4717309

[ref54] Singh B. Salaria N. Thakur K. Kukreja S. Gautam S. Goutam U. (2019). Functional genomic approaches to improve crop plant heat stress tolerance. F1000Res 8:1721. doi: 10.12688/f1000research.19840.1, PMID: 31824669 PMC6896246

[ref55] Song M. Yin Y. He Z. Tong C. Zhu L. Gao. (2019). Genome-wide characterization and expression profiling of squamosa promoter binding protein-like (SBP) transcription factors in wheat (*Triticum aestivum* L.). Agronomy 9, 527–558. doi: 10.3390/agronomy9090527

[ref56] Wang Y. Yu Y. Huang M. Gao P. Chen H. Liu M.. (2020). Transcriptomic and proteomic profiles of II YOU 838 (*Oryza sativa*) provide insights into heat stress tolerance in hybrid rice. PeerJ 8:e8306. doi: 10.7717/peerj.8306, PMID: 32117601 PMC7039125

[ref57] Xing G. Li J. Li W. Lam S. M. Yuan H. Shui G.. (2021). AP2/ERF and R2R3-MYB family transcription factors: potential associations between temperature stress and lipid metabolism in *Auxenochlorella* protothecoides. Biotechnol. Biofuels 14:22. doi: 10.1186/s13068-021-01881-6, PMID: 33451355 PMC7811268

[ref58] Xu L. Feng G. Yang Z. Xu X. Huang L. Yang Q.. (2020). Genome-wide AP2/ERF gene family analysis reveals the classification, structure, expression profiles and potential function in orchardgrass (*Dactylis glomerata*). Mol. Biol. Rep. 47, 5225–5241. doi: 10.1007/s11033-020-05598-x, PMID: 32577992

[ref59] Yamaguchi-Shinozaki K. Shinozaki K. (2006). Transcriptional regulatory networks in cellular responses and tolerance to dehydration and cold stresses. Annu. Rev. Plant Biol. 57, 781–803. doi: 10.1146/annurev.arplant.57.032905.105444, PMID: 16669782

[ref60] Ye J. Yang X. Hu G. Liu Q. Li W. Zhang L.. (2020). Genome-wide investigation of heat shock transcription factor family in wheat (*Triticum aestivum* L.) and possible roles in anther development. Int. J. Mol. Sci. 21, 609–629. doi: 10.3390/ijms21020608, PMID: 31963482 PMC7013567

[ref61] Zhao Y. Ma R. Dongliang X. Bi H. Xia Z. Peng H. (2019). Genome-wide identification and analysis of the AP2 transcription factor gene family in wheat (*Triticum aestivum* L.). Front. Plant Sci. 10:1286. doi: 10.3389/fpls.2019.01286, PMID: 31681381 PMC6797823

[ref62] Zhou L. Yarra R. (2021). Genome-wide identification and characterization of AP2/ERF transcription factor family genes in oil palm under abiotic stress conditions. Int. J. Mol. Sci. 22, 2821–2836. doi: 10.3390/ijms22062821, PMID: 33802225 PMC8000548

