# Supplementary material for: Genome-Wide Analysis of AP2/ERF Superfamily Genes in Contrasting Wheat Genotypes Reveals Heat Stress-Related Candidate Genes
Source: Front Plant Sci. 2022 Apr 13;13:853086. doi: 10.3389/fpls.2022.853086 (PMC9044922; doi:10.3389/fpls.2022.853086)
Supplement: Supplementary file 1 [file Data_Sheet_1.docx]

Supplementary Material

Supplementary Figures


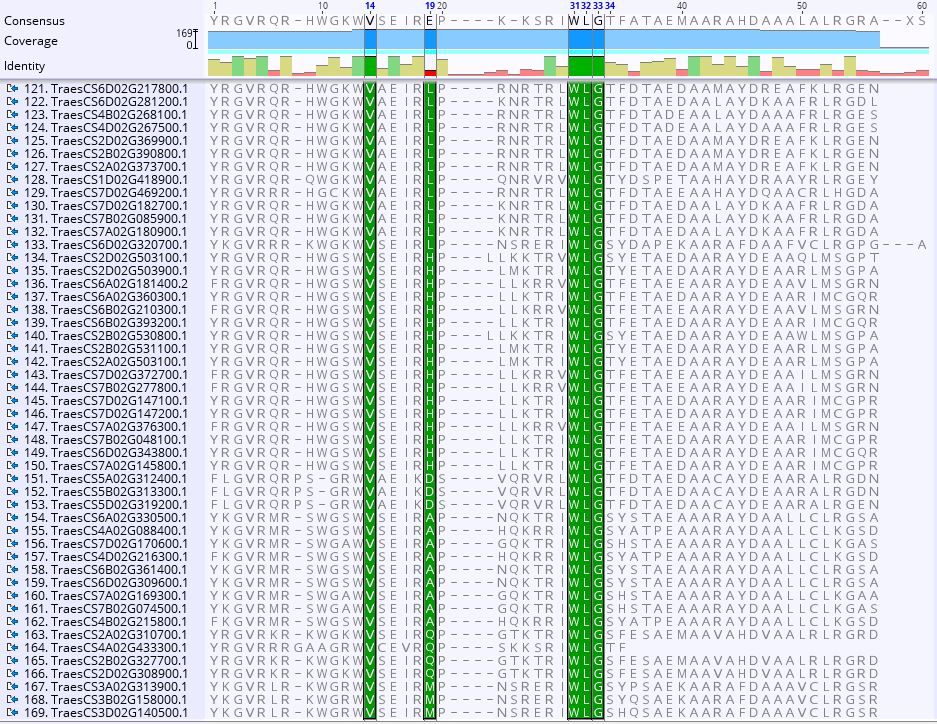

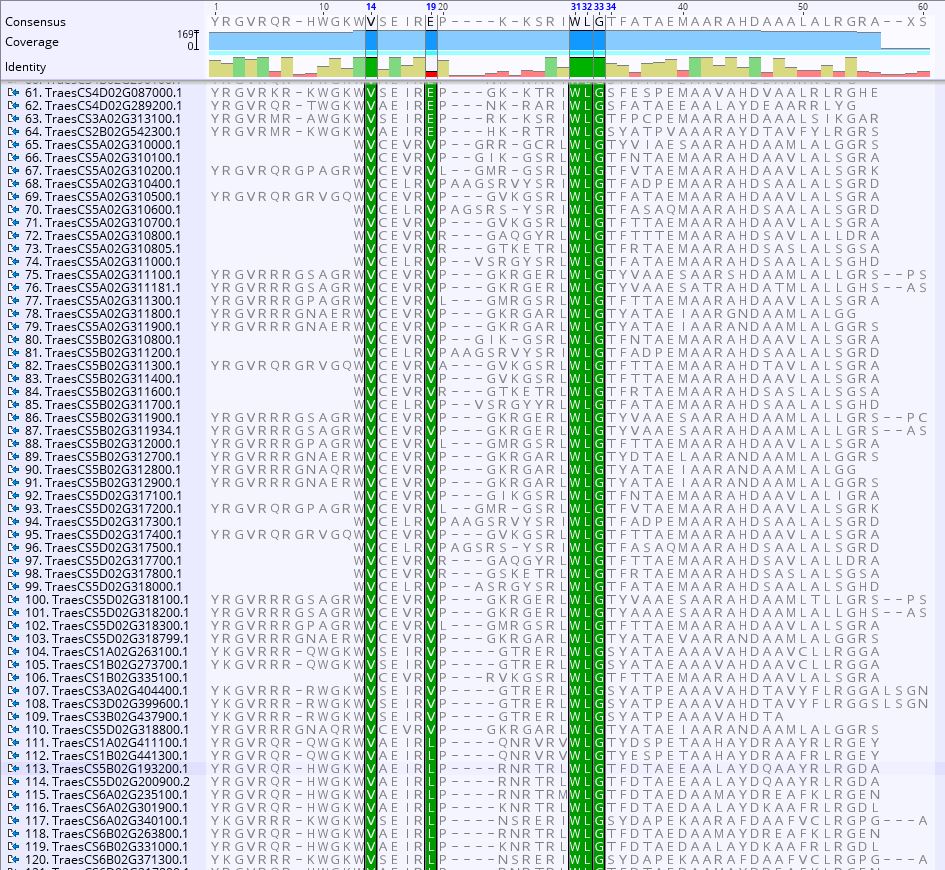

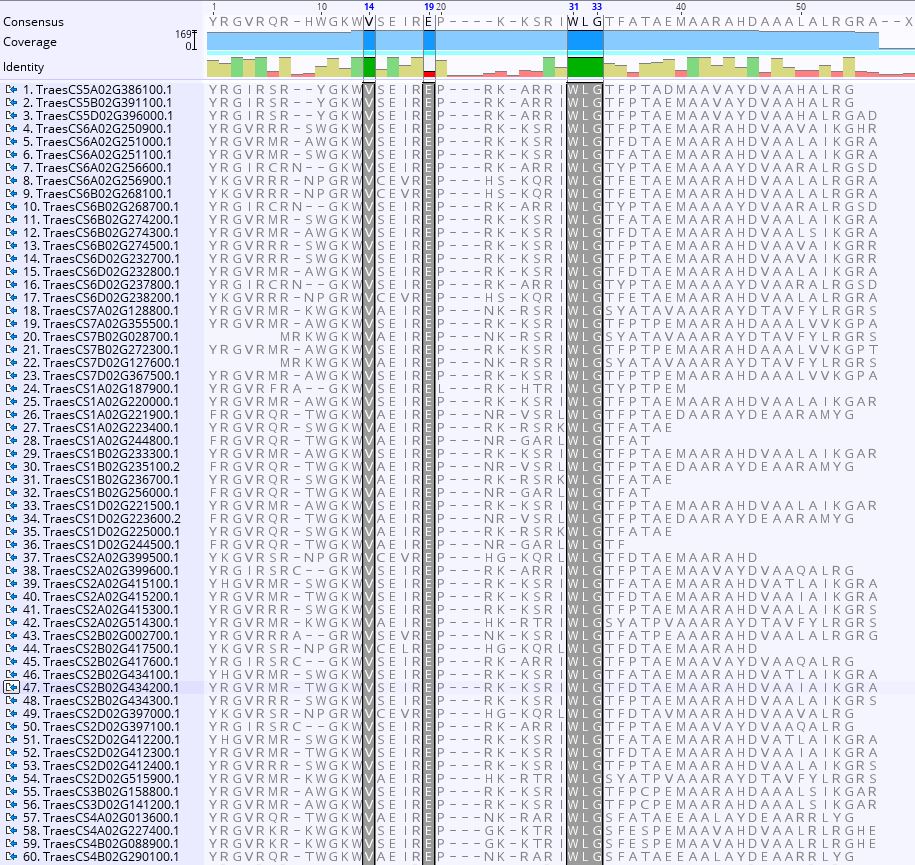


**Supplementary Figure 1.** Multiple alignment of domain sequences of 169 TaDREB family TF genes showing conserved amino acid at 14^th^ and 19^th^ position. Sixty-four TaDREB genes show V (valine) at 14^th^ and E (glutamic acid) at 19^th^ positions and the remaining 105 showing at least one of these two amino acids conserved while the other having E replaced by H, L, A, V, Q or M.

.


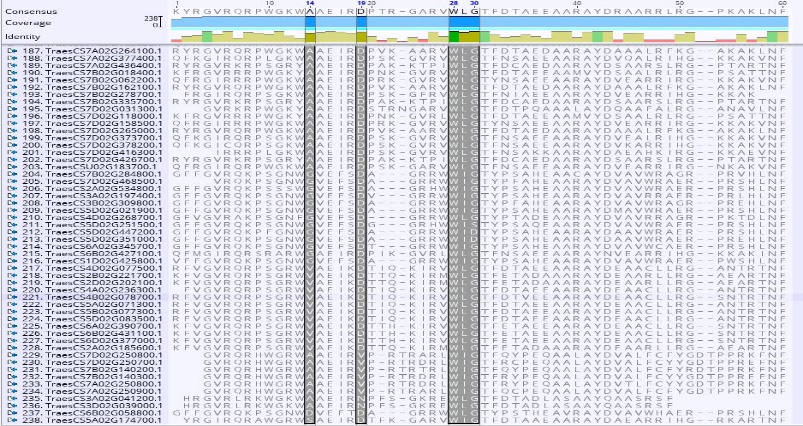

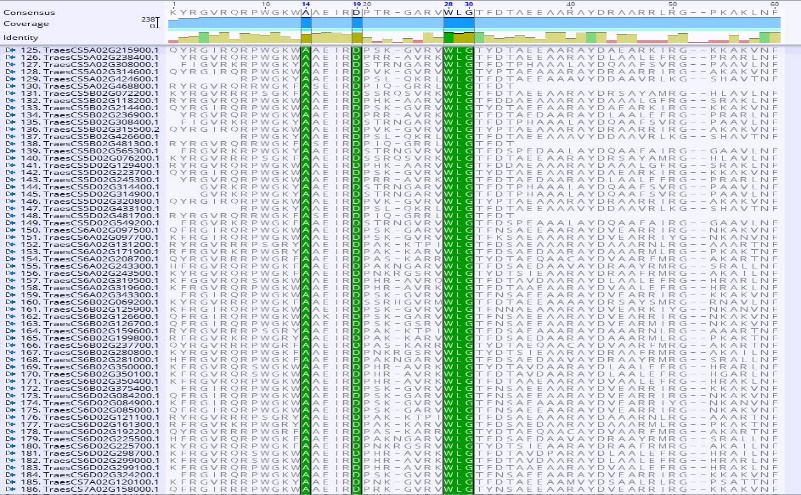

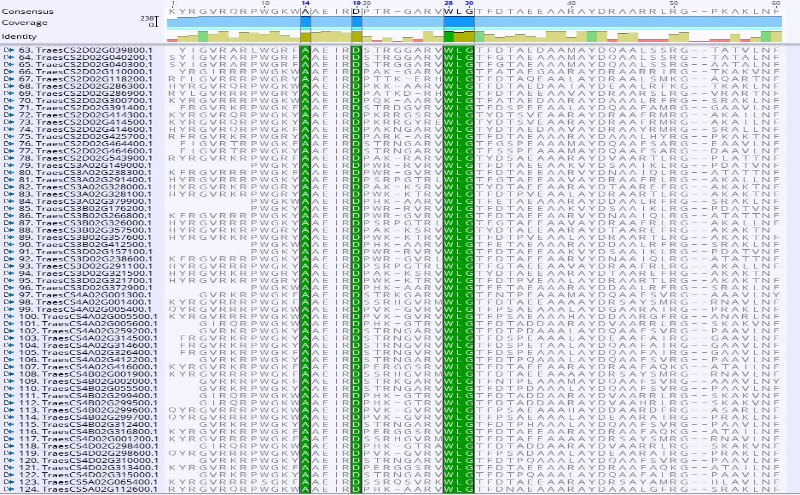

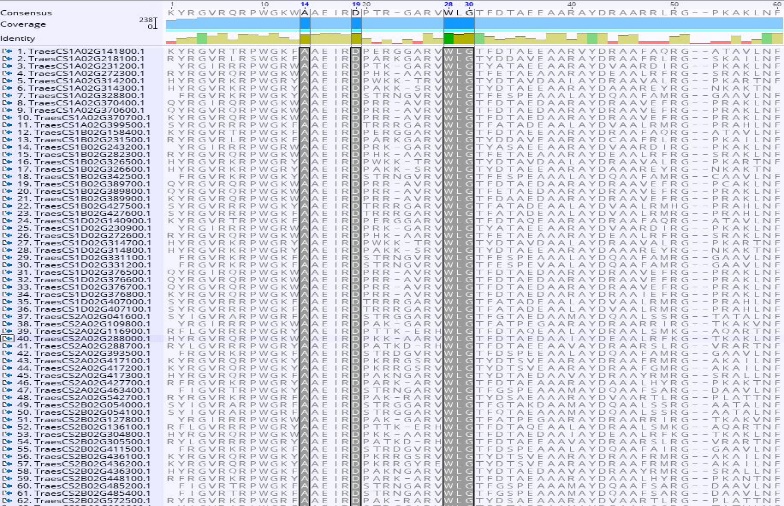


**Supplementary Figure 2.** Multiple alignment of domain sequences of 238 TaERF family TF genes showing conserved amino acid at 14^th^ and 19^th^ position. Two hundred and three TaERF genes show A: (alanine) at 14^th^ and D (aspartic acid) at19^th^ positions, and the remaining 35 showing at least one of these two amino acids conserved while the other having either A replaced by D, G, S, V, or D replaced by V, N.


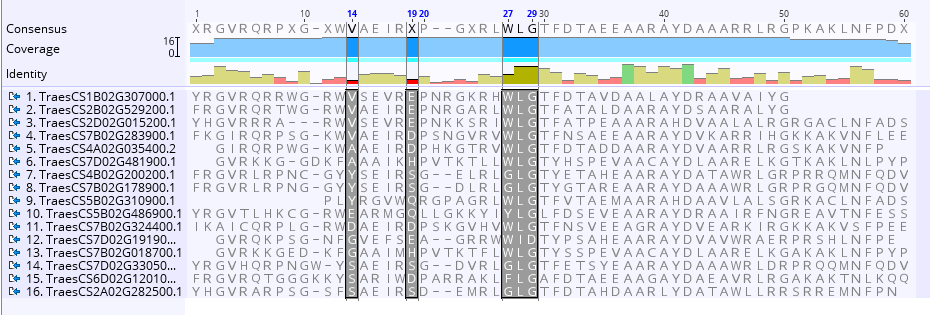


**Supplementary Figure 3:** Multiple alignment of domain sequence 16 TaERF/TaDREB like TF genes. Nine genes with either V at 14^th^ position or D at 19^th^ position conserved and 7 genes with none of these 4 amino acids (V, E, A, D) conserved at 14^th^ or 19^th^ position, but carrying a distinct AP2 domain.

| A1 A2 A3  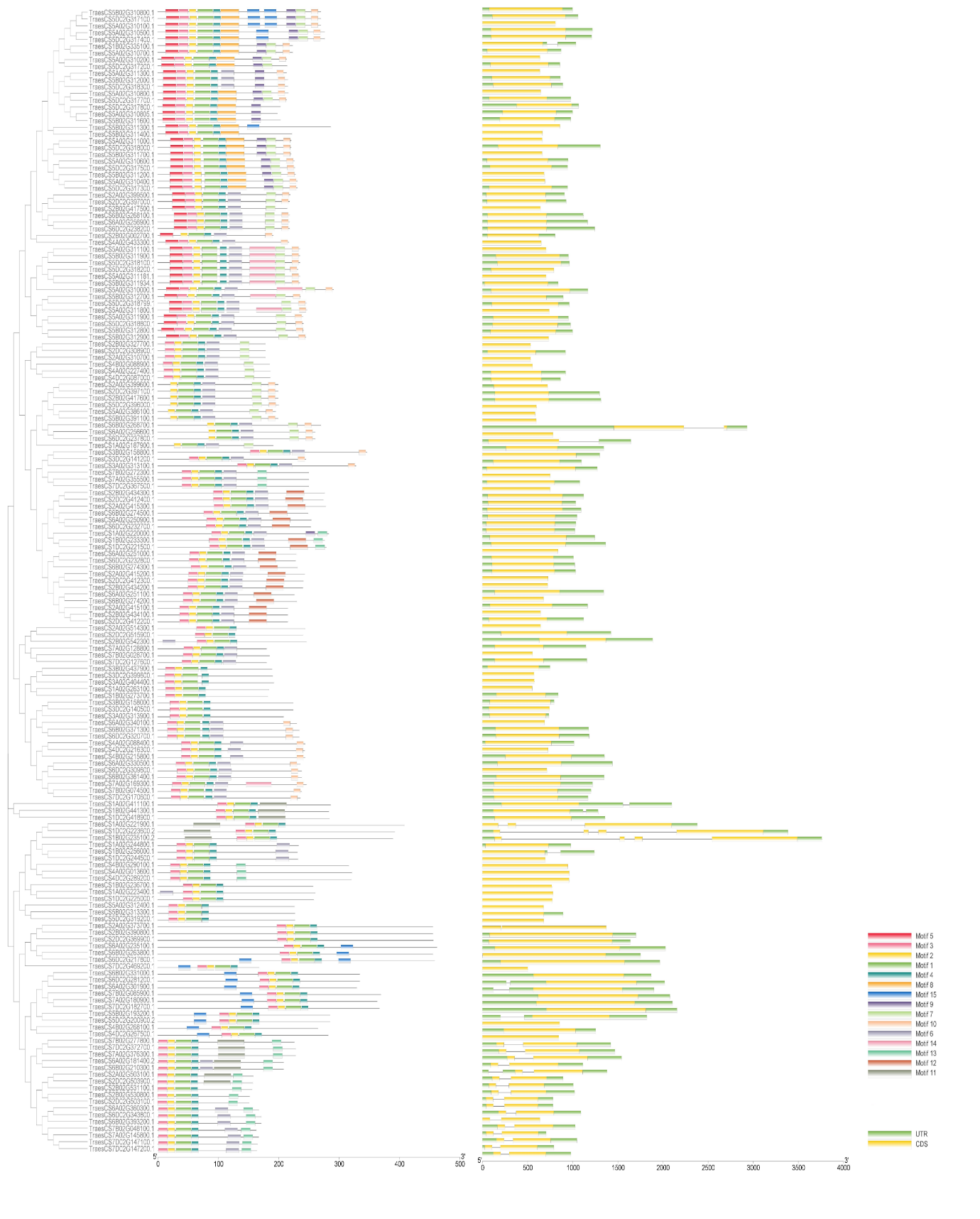 |
| --- |
| A |

| B1 B2 B3  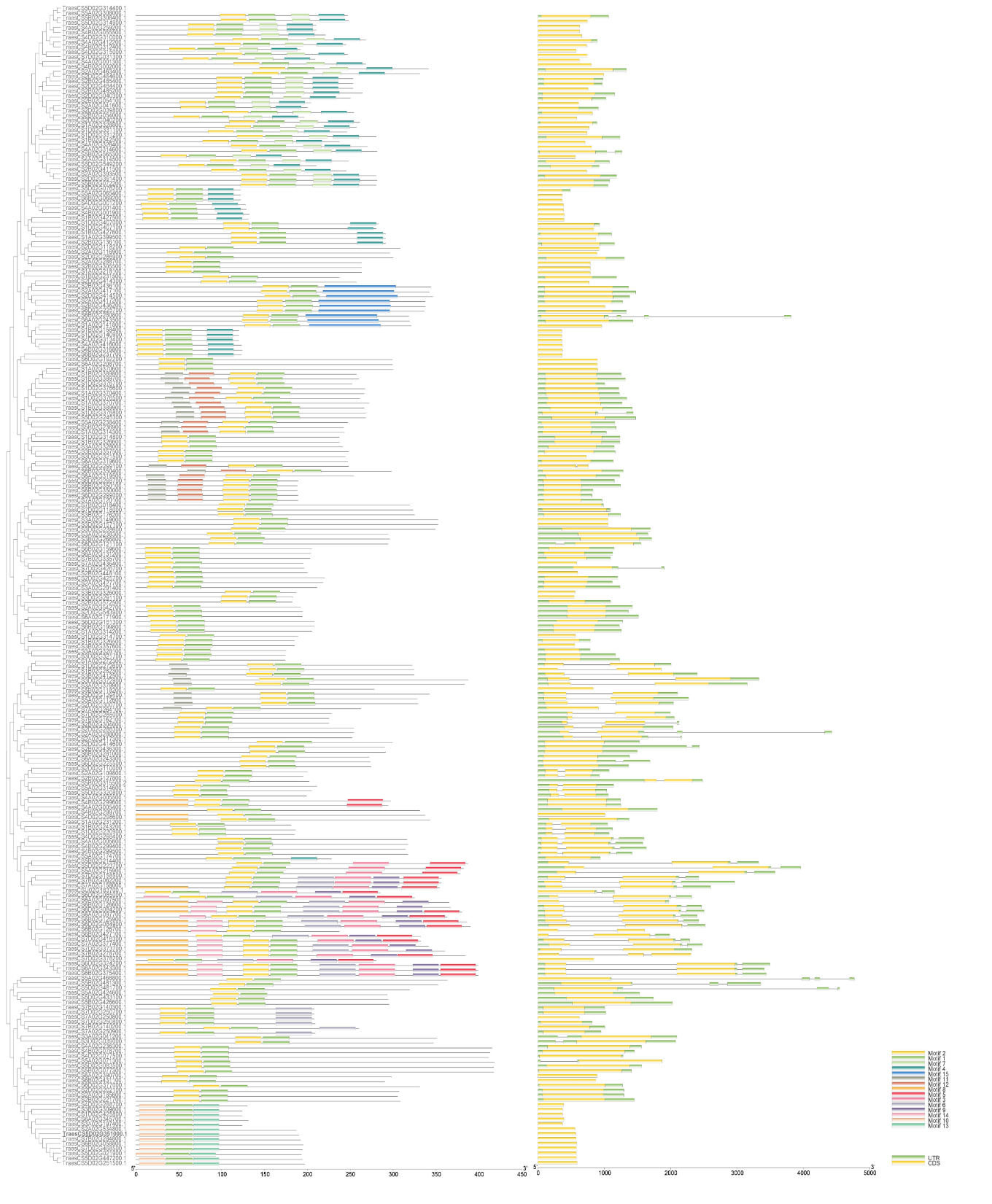 |
| --- |
| B |

| \| - C1 C2 C3 \| - D1 D2 D3 \| \| --- \| --- \| \| - **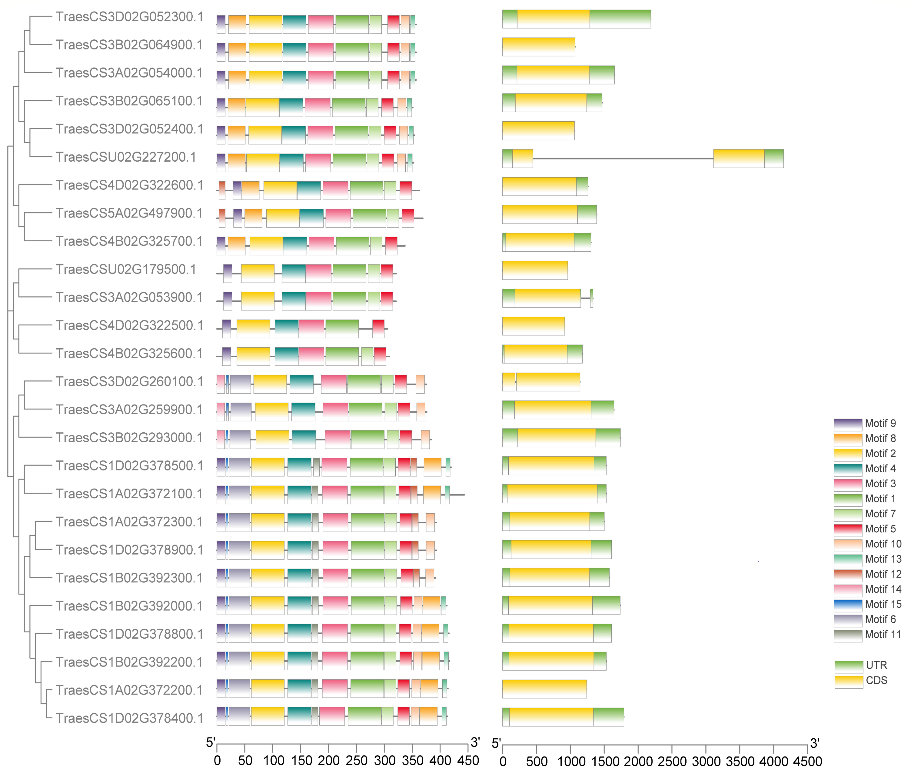** \| - **** \| \| - **C** \| - **D** \| |
| --- | --- | --- | --- | --- | --- | --- |
| **Supplementary Figure 4.** Distribution of conserved motifs, exon-intron-UTR structure, and phylogenetic grouping of 351 TaAP2/ERF super-family TF genes.   1. Phylogeny (A1), conserved motif distribution (A2) and gene structure (A3) in 26 TaDREB genes; 2. Phylogeny (B1), conserved motif distribution (B2) and gene structure (B3) in 66 TaERF genes; 3. Phylogeny (C1), conserved motif distribution (C2) and gene structure (C3) of 169 TaRAV genes and 4. Phylogeny (D1), conserved motif distribution (D2) and gene structure (D3) of 238 TaAP2 genes.   The phylogenetic trees were constructed using MEGAX with bootstrap values of 1000 repeats, motif composition was analysed using online MEME tool and gene structure analysis was done by GSDS online server. In motif graph, the coloured blocks represent the position of motifs in corresponding proteins coding TF genes, block size indicates the length of motifs and grey lines connecting the coloured bocks represents the non-conserved sequences. In gene structure graph, the yellow blocks are exon, green blocks are UTR and grey lines are introns. The relative position of each motifs and exon, intron and UTR can be determined with the help of scale displayed just below corresponding graph. |


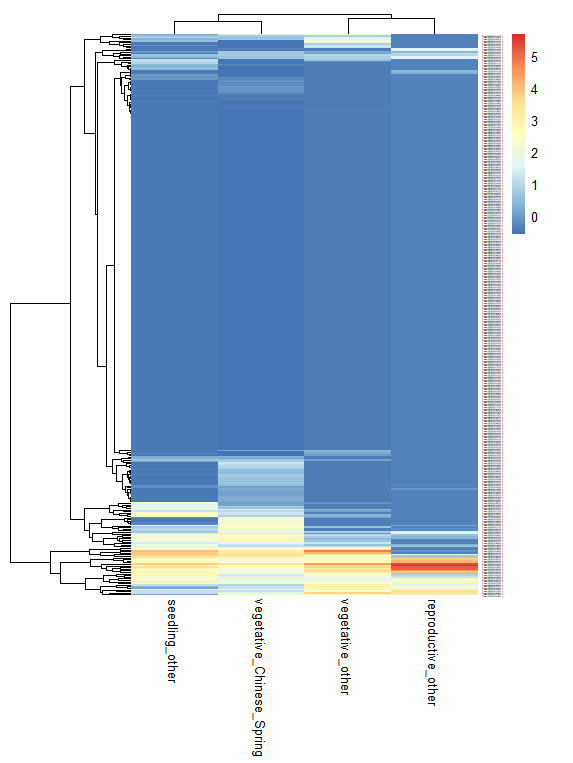


**Supplementary Figure 5:** The expression pattern profiling of 351 TaAP2/ERF TF genes under abiotic stress using publicly available transcriptome data. The intensity of color shows the level of gene expression, where blue color shows the least expressed gene, and the red color shows the highest expressed gene. The genes are clustered into two major clusters based on their expression values forming a bigger cluster of relatively low expressed genes (91%) and a smaller cluster of higher expressed genes (9%) under abiotic stress.


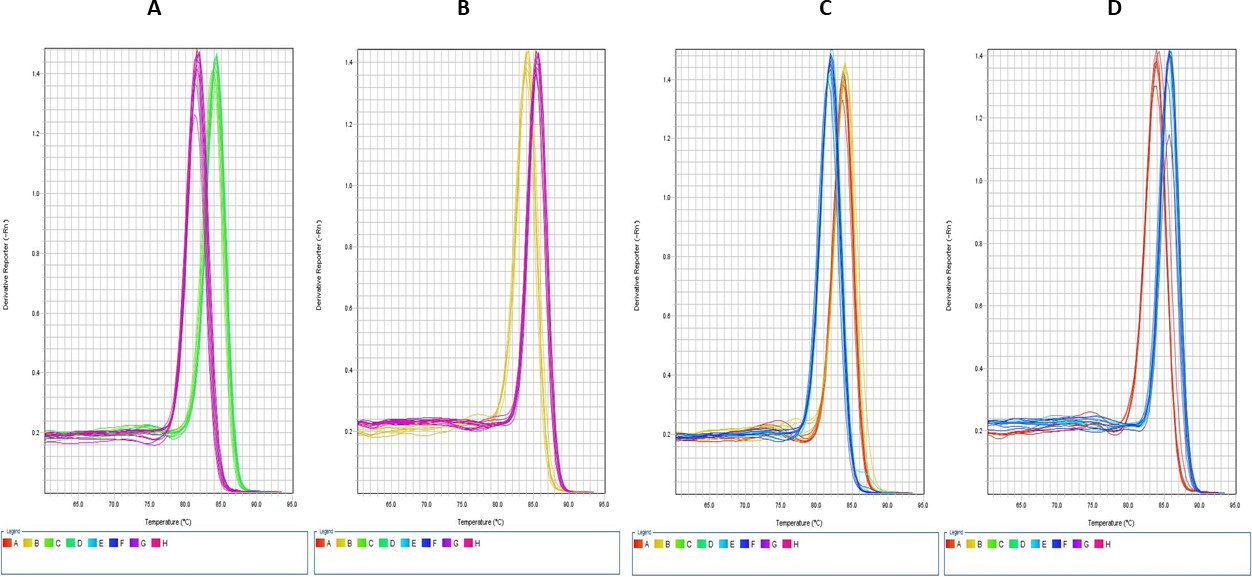


**Supplementary Figure 6:** qRT-PCR melting curve showing single peak for the TraesCS4D02G298600.1 and TraesCS5B02G193200.1 genes. A) TraesCS4D02G298600.1 gene conferring heat tolerance in heat-tolerant genotype (purple sample peak against green reference peak); and B) TraesCS5B02G193200.1 gene conferring heat tolerance in heat-tolerant genotype (purple sample peak against yellow reference peak); C) TraesCS4D02G298600.1 gene conferring heat susceptibility in heat-susceptible genotype (blue sample peak against yellow reference peak); and D) TraesCS5B02G193200.1 gene conferring heat susceptibility in heat-susceptible genotype (blue sample peak against red reference peak).
